# AI and Interventional Radiology: A Narrative Review of Reviews on Opportunities, Challenges, and Future Directions

**DOI:** 10.3390/diagnostics15070893

**Published:** 2025-04-01

**Authors:** Andrea Lastrucci, Nicola Iosca, Yannick Wandael, Angelo Barra, Graziano Lepri, Nevio Forini, Renzo Ricci, Vittorio Miele, Daniele Giansanti

**Affiliations:** 1 Department of Allied Health Professions, Azienda Ospedaliero-Universitaria Careggi, 50134 Florence, Italy; andrea.lastrucci@unifi.it (A.L.); ioscan@aou-careggi.toscana.it (N.I.); wandaely@aou-careggi.toscana.it (Y.W.); barraa@aou-careggi.toscana.it (A.B.); riccire@aou-careggi.toscana.it (R.R.); 2 Unità Sanitaria Locale Umbria 1, Via Guerriero Guerra 21, 06127 Perugia, Italy; graziano.lepri@uslumbria1.it; 3 Dipartimento di Medicina e Chirurgia, Universita’ degli Studi di Perugia, Piazzale Settimio Gambuli, 1, 06129 Perugia, Italy; nevio.forini@unipg.it; 4 Department of Experimental Clinical and Biomedical Sciences, University of Florence, 50134 Florence, Italy; vmiele@sirm.org; 5 Department of Radiology, Careggi University Hospital, 50134 Florence, Italy; 6 Centro TISP, Istituto Superiore di Sanità, Via Regina Elena 299, 00161 Roma, Italy

**Keywords:** radiology, interventional radiology, artificial intelligence, machine learning, deep learning

## Abstract

The integration of artificial intelligence in interventional radiology is an emerging field with transformative potential, aiming to make a great contribution to the health domain. This overview of reviews seeks to identify prevailing themes, opportunities, challenges, and recommendations related to the process of integration. Utilizing a standardized checklist and quality control procedures, this review examines recent advancements in, and future implications of, this domain. In total, 27 review studies were selected through the systematic process. Based on the overview, the integration of artificial intelligence (AI) in interventional radiology (IR) presents significant opportunities to enhance precision, efficiency, and personalization of procedures. AI automates tasks like catheter manipulation and needle placement, improving accuracy and reducing variability. It also integrates multiple imaging modalities, optimizing treatment planning and outcomes. AI aids intra-procedural guidance with advanced needle tracking and real-time image fusion. Robotics and automation in IR are advancing, though full autonomy in AI-guided systems has not been achieved. Despite these advancements, the integration of AI in IR is complex, involving imaging systems, robotics, and other technologies. This complexity requires a comprehensive certification and integration process. The role of regulatory bodies, scientific societies, and clinicians is essential to address these challenges. Standardized guidelines, clinician education, and careful AI assessment are necessary for safe integration. The future of AI in IR depends on developing standardized guidelines for medical devices and AI applications. Collaboration between certifying bodies, scientific societies, and legislative entities, as seen in the EU AI Act, will be crucial to tackling AI-specific challenges. Focusing on transparency, data governance, human oversight, and post-market monitoring will ensure AI integration in IR proceeds with safeguards, benefiting patient outcomes and advancing the field.

## 1. Introduction

Interventional radiology (IR) based on the Collins Dictionary [1] is defined as “an application of radiology that enables minimally invasive surgery to be performed with the aid of simultaneous radiological imaging of the field of operation within the body”. According to the Oxford Dictionary [2], it involves “The performance of therapeutic or diagnostic procedures under the control of an appropriate imaging technique. Guidance is commonly by X-ray fluoroscopy, ultrasound, or computerized tomography, and recently also by magnetic resonance imaging. Procedures commonly performed include the placing of vascular catheters, drainage of fluid collections or abscesses, stenting of obstructions to the gastrointestinal tract or blood vessels, embolization, cryotherapy, and radiofrequency ablation. Imaging is also used in many forms of minimally invasive surgery.” Interventional radiology is a relatively recent discipline that has revolutionized the way many diseases are treated. It combines the expertise of radiology with other specialties, such as surgery, oncology, and urology, offering less invasive solutions compared to traditional procedures. The key advantage of this technique is the ability to perform image-guided interventions, reducing recovery times and minimizing risks for the patient [3].

### 1.1. The Scope and Implications of Interventional Radiology

Interventional radiology (IR) is a transformative medical field that has seen rapid advancements and is applied across numerous disciplines. The applications and implications of this specialty include the following key areas.

#### 1.1.1. Key Support in Surgery and Oncology

One of the most valuable applications of IR is in controlling hemorrhages. Through embolization techniques, blood vessels can be sealed from the inside, avoiding the need for more invasive surgical procedures. This is particularly beneficial in the postoperative period when complications such as bleeding might otherwise require reoperation.

IR has also made significant strides in cancer treatment [4]. Using image-guided probes (via CT scans, MRI, or ultrasound), doctors can precisely target tumors and destroy them from within. Different techniques are employed depending on the type of tumor; some use heat—emitted by radiofrequency or microwaves—to “burn” cancer cells, while others utilize extreme cold (−80 °C) to freeze and destroy them.

In certain cases, IR even enables the treatment of tumors once considered inoperable. Innovative techniques such as radioembolization, which involves injecting radioactive microspheres directly into the tumor, can slow or halt disease progression. Another promising approach is the use of genetically modified viruses that selectively attack cancer cells while leaving healthy tissue unharmed.

#### 1.1.2. Beyond Oncology: Interventional Radiology in Medical Specialties

Interventional radiology extends far beyond cancer treatment. It is widely used in managing vascular diseases, including angioplasty to reopen narrowed arteries or veins and embolization to treat aneurysms and arteriovenous malformations [5].

IR is also proving highly effective in treating liver and kidney conditions, offers minimally invasive alternatives that can replace traditional surgical interventions. In liver treatment, for example, transarterial chemoembolization (TACE) allows for the direct injection of chemotherapy drugs into liver tumors, minimizing systemic side effects.

#### 1.1.3. Pain Management and Contributions to Quality of Life

Interventional radiology plays a crucial role in pain management. Techniques such as vertebroplasty and kyphoplasty stabilize vertebral fractures—often caused by osteoporosis or metastases—providing immediate pain relief [6].

Additionally, nerve ablation is used to treat chronic pain in cancer patients and those with other debilitating conditions.

#### 1.1.4. Applications in Gynecology, Urology, and Gastroenterology

IR also offers innovative solutions in gynecology and urology [7]. For instance, uterine fibroid embolization effectively reduces fibroids without the need for surgery, while prostatic artery embolization is a promising treatment for benign prostatic hyperplasia.

In gastroenterology, stents are used to treat esophageal, gastric, and intestinal strictures, significantly improving the quality of life of patients suffering from tumor-related or other types of obstructions. Additionally, abscesses in the abdomen or pancreatic cysts can be safely and minimally invasively drained.

#### 1.1.5. The Importance of Vascular Access

Another crucial aspect of interventional radiology is vascular access management. The placement of central venous catheters such as peripherally inserted central catheter lines or port-a-caths is essential for patients requiring long-term therapies, such as chemotherapy or parenteral nutrition [8]. Thanks to imaging guidance, these procedures are performed with high precision and safety.

### 1.2. A Continuously Evolving Future of Interventional Radiology

With ongoing technological advancements, interventional radiology is expanding its scope, offering increasingly effective and minimally invasive solutions. These new techniques not only improve patients’ quality of life but also often serve as safer and more efficient alternatives to traditional surgeries. The future of this discipline is continuously evolving, with new approaches that promise to make medicine even more precise, effective, and patient-centered, accompanied by a strong specialization of the professionals involved [9].

One of the most exciting developments in IR [9] is the integration of artificial intelligence (AI), robotics, augmented reality (AR), and virtual reality (VR). AI is revolutionizing the field by assisting with real-time image analysis, optimizing procedural planning, and enhancing decision-making accuracy. For example, AI-powered image analysis can detect subtle anomalies in real-time, such as small tumors or vessel blockages, allowing for faster and more accurate diagnoses during procedures. Robotics increases precision by minimizing human error and even enables remote interventions, making IR more accessible.

AR and VR technologies play a critical role in training and real-time guidance, offering immersive environments that improve catheter navigation and treatment delivery during complex procedures. For instance, AR systems can overlay 3D images of a patient’s anatomy onto real-time X-ray or CT scans, guiding the physician’s movements during catheter insertions, biopsies, or tumor ablations. When integrated with AI, AR can provide dynamic, real-time adjustments to these images, enhancing precision during delicate procedures. VR, on the other hand, allows for hands-on training in a simulated environment, where medical professionals can practice complex interventions without risk to patients, accelerating skill development.

As these technologies continue to advance, IR is poised to become even more adaptable and efficient, reducing invasiveness and improving patient outcomes. The future of IR will be shaped by a seamless fusion of human expertise and cutting-edge technology, with AI at the forefront of this transformation. However, the integration of these technologies also brings challenges that require careful monitoring and continual adaptation to ensure the best possible impact on patient care.

AI acts as a mediator [10], assisting in image analysis, optimizing procedural planning, and improving real-time decision-making. Robot-assisted interventions increase accuracy, reducing human error and enabling remote procedures. AR and VR technologies offer immersive training environments and real-time guidance during complex interventions, allowing for more precise catheter navigation and treatment delivery. As these innovations continue to evolve, interventional radiology is set to become even more adaptive, personalized, and minimally invasive, with AI playing a significant role. However, this integration introduces challenges and ongoing needs that must be monitored closely to ensure its successful implementation and optimal impact on patient care [9,10].

### 1.3. Purpose

It is therefore important to analyze both the evolution of the integration between AI and interventional radiology (IR) as well as the ongoing implications and challenges. An overview of the published reviews allows us to bring this out in a comprehensive manner. The general aim of this study is, therefore, to provide a narrative review of reviews (NRR) with the purpose of analyzing the state of integration of AI with interventional radiology, with the following specific objectives:

- Analyze the overall bibliometric trends in the field: This study aims to provide a comprehensive bibliometric analysis of research output, focusing on trends and developments over time.
- Identify established themes and categories: Identify key areas of focus in reviews, such as AI applications, diagnostic accuracy, automation, decision support systems, and workflow optimization.
- Examine Opportunities and Challenges: Explore the potential benefits and challenges of integrating AI into interventional radiology, such as improving procedural precision, reducing human error, enhancing patient outcomes, and addressing issues like data privacy, regulatory concerns, and technology adoption barriers.

## 2. Methodology Overview

The methodology comprises the following two main approaches: a narrative review of reviews and a bibliometric analysis. The narrative review synthesizes existing literature on digital cytopathology, focusing on advancements, challenges, and the role of artificial intelligence (AI). The bibliometric analysis examines historical research trends in digital cytopathology, particularly over the last decade with an emphasis on AI-related studies.

These two methods were chosen to both make an in-depth synthesis of research in the field of AI and interventional radiology and to statistically evaluate their development over time.

### 2.1. Narrative Review Selection and Qualification Process

#### 2.1.1. Checklist and Qualification

The narrative review followed a structured selection process based on the ANDJ standardized narrative checklist. Targeted searches were conducted using PubMed and Scopus with predefined composite search keys.

A qualification methodology was employed, using quality parameters to determine study inclusion and a predefinite procedure (see the Algorithm 1 below).
**Algorithm 1:** Selection Process for the NRR**Define search query:**○*“(interventional radiology[Title/Abstract]) AND ((neural network[Title/Abstract]) OR (Artificial Intelligence[Title/Abstract]) OR (deep learning[Title/Abstract]) OR (ANN[Title/Abstract]) OR (GAN[Title/Abstract]))”*2.**Conduct searches** in PubMed and Scopus using the defined query.3.**Select relevant studies** from peer-reviewed journals that focus on the field priority, such as recent reviews that assess prior studies and reviews providing broad analyses, integrating findings from previous works and with a focus on AI applications in line with the journal topic (articles more focused on computer sciences were excluded on the basis that they were not in line the focus of the journal).4.**Evaluate each study** based on the following parameters:
○**N1**: Clear rationale in the introduction.○**N2**: Appropriate research design.○**N3**: Clearly described methodology.○**N4**: Well-presented results.○**N5**: Conclusions justified by results.○**N6**: Disclosure of conflicts of interest.5.**Assign scores** to parameters **N1–N5** (scale of 1–5).6.**Assess N6** using a binary Yes/No measure.7.**Preselect studies** meeting the following criteria:
○**N6 = “Yes”** (conflict of interest disclosed).○**N1–N5 scores > 3** (ensuring methodological rigor).
8.**Include preselected studies** in the final synthesis.

#### 2.1.2. Assessment Process

Each study included in the analysis was reviewed by two initial assessors (A.L and D.G.). These assessors were responsible for evaluating the studies based on their focus on the integration of artificial intelligence (AI) in interventional radiology and according to predefined criteria. The assessment criteria included Clarity of Rationale, Study Design Appropriateness, Methodological Rigor, Result Presentation, Justification of Conclusions, and Disclosure of Conflicts of Interest. Each criterion was scored on a predefined scale to provide a quantitative measure of the quality and relevance of the analyzed studies. The primary assessors independently reviewed the studies and assigned scores to each parameter, ensuring that each study was evaluated against uniform standards. This dual-assessment approach was designed to enhance the reliability of the review by incorporating different perspectives and reducing the likelihood of individual bias influencing the evaluation process.

In cases where the two initial assessors disagreed on the scores or the inclusion of a study, a third assessor chosen with a rotation criterion (either G.L. or Y.W.) was involved to adjudicate the decision. This third-party assessment played a crucial role in resolving conflicts and ensuring that final decisions were fair and well-justified. The additional scrutiny provided by the third assessor helped balance differing opinions and reinforced the integrity of the review process.

The multi-assessor approach was implemented to minimize bias and ensure a rigorous and balanced evaluation of the literature. By integrating multiple viewpoints and providing a structured mechanism for resolving disagreements, the review aimed to offer a comprehensive and objective assessment of AI applications in interventional radiology.

#### 2.1.3. Managing Bias in the Review

To ensure the objectivity and methodological rigor of the review, several strategies were implemented to manage and minimize bias throughout the assessment process:

*Diverse Assessors*:

Each study was reviewed by two assessors with different backgrounds, selected to ensure a variety of perspectives. The inclusion of assessors with expertise in both interventional radiology and AI reduced the risk of individual biases influencing the evaluation.

*Clear Assessment Criteria*:

The studies were analyzed using predefined criteria, including Clarity of Rationale, Study Design Appropriateness, Methodological Rigor, Result Presentation, Justification of Conclusions, and Disclosure of Conflicts of Interest. Furthermore, data were presented based on a standardized checklist, reducing the risk of subjective interpretation.

*Scoring System*:

Each parameter was rated on a scale from 1 to 5, while the disclosure of conflicts of interest was assessed using a binary evaluation (Yes/No). This quantitative approach allowed for consistent evaluations across studies and provided a transparent mechanism for comparing study quality.

*Independent Review*:

The primary assessors reviewed the studies independently, assigning scores without prior discussion. This independence helped ensure that individual judgments were based solely on the study’s merit and predefined criteria, minimizing groupthink or shared biases.

*Dispute Resolution*:

In cases where the two assessors disagreed on scores or study inclusion, a third assessor was consulted to provide an impartial judgment. This third-party adjudication helped resolve conflicts fairly, offering an additional level of scrutiny and ensuring balanced decision-making.

*Structured Mechanism for Disagreements*:

The process for resolving disagreements was formalized and structured. The third assessor reviewed the initial evaluations and provided a reasoned judgment to reconcile differences. This structured approach ensured that conflicts were systematically addressed and that final decisions were based on a comprehensive evaluation.

*Transparency*:

The use of a standardized checklist for data presentation and a clear scoring system enhanced transparency in the assessment process. By documenting the criteria and scoring rationale, the review process became more traceable and reproducible, reducing the potential for undisclosed biases.

By incorporating these strategies, this review aimed to offer a thorough and balanced evaluation of the literature. The multi-assessor approach, combined with structured criteria and a formal dispute resolution mechanism, was designed to minimize bias and enhance the reliability and objectivity of the review process.

#### 2.1.4. Selected Studies

**Figure 1:**
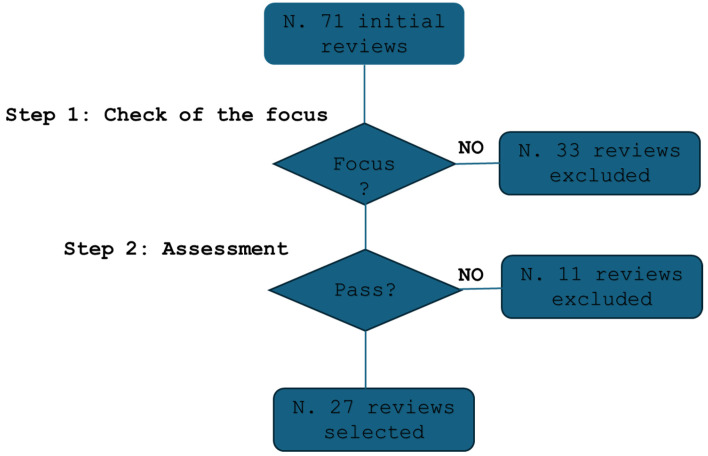
Details on the process of study selection.

Figure 1 illustrates that the initial search yielded a total of 71 reviews. From these, 33 studies were excluded due to their lack of direct focus. Following the evaluation according to the methodology described above, 27 review studies were retained [11,12,13,14,15,16,17,18,19,20,21,22,23,24,25,26,27,28,29,30,31,32,33,34,35,36,37] for further consideration, while 11 studies were excluded.

### 2.2. Bibliometric Analysis Methodology

The methodological approach involved a multi-step analysis combining quantitative, statistical, graphical, and narrative methods. First, a systematic search was conducted on PubMed using specific keywords related to artificial intelligence (AI) and interventional radiology (IR). The quantitative analysis focused on the total number of publications, identifying trends in publication frequency and categorizing articles into primary research and review types. The statistical analysis was used to calculate trends across the entire period, as well as focusing on the last 10 and 5 years. The data were segmented into these timeframes to identify patterns in the publication frequency and to examine the rate of growth in AI-related research within interventional radiology (IR). This analysis helped highlight periods of significant acceleration, particularly in the last five years, and allowed for comparison between broader trends and more recent developments. Graphical representations, such as trends over time and distribution of study types, were generated to visualize the growth of AI research in IR, especially in the last five years. Finally, a narrative approach was used to interpret the results, considering the factors influencing this increase, such as advancements in AI technologies, the impact of the COVID-19 pandemic, increased funding, and the regulatory and clinical adoption of AI in medical practices.

## 3. Results

The study results have been organized into three main sections to provide a structured and in-depth overview.

*Section 3.1*: Focuses on bibliometric trends in this field by analyzing the progression of publication volumes over time, with a particular emphasis on the last five. This offers insights into the evolution of, and growing interest in, the topic.

*Section 3.2*: Examines the selected review studies, which focus on the application of AI in IR. This section explores emerging themes and provides a categorization to better understand the key areas of research and innovation.

*Section 3.3*: Based on the findings from the analyzed studies, it identifies emerging opportunities and highlights barriers that require further exploration and development to drive significant advancements in the field.

This systematic approach provides a clear and comprehensive view of the state of the art, current trends, and future prospects in the application of AI to assistive technologies in healthcare.

### 3.1. Trends

**Figure 2:**
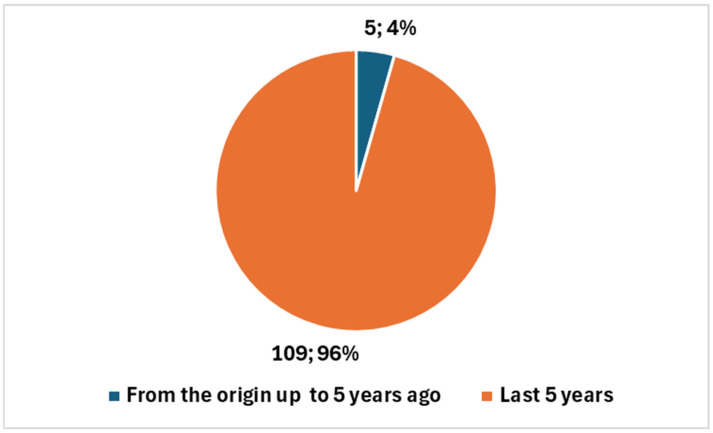
Distribution of AI-related publications in interventional radiology—comparison between the last five years and earlier periods.

A comprehensive literature search conducted on PubMed (refer to Key 1 in Box 1) provides insight into the evolution of research in interventional radiology. Since 1978, a total of **10,947 studies** have been published in this field, reflecting decades of scientific advancements and clinical innovations. However, when refining the search criteria to focus specifically on the integration of **artificial intelligence** (**AI**) **in interventional radiology** (see Key 2 in Box 1), the number of studies drops dramatically to just **114**. This figure represents a mere **1.04%** of all published research in interventional radiology, underscoring that AI is still an emerging area within this specialty (Figure 2).

A closer examination of the timeline of AI-related publications reveals a **notable concentration of research in recent years**. Out of the **114 AI-focused studies**, **104** (**over 91%**) **were published in the last five years**, highlighting a significant surge in interest and scientific output in this domain. This period has been profoundly shaped by the **COVID-19 pandemic**, which accelerated the adoption of **digital health technologies**, **automation**, **and AI-driven innovations** across various medical disciplines. The pandemic created both challenges and opportunities, prompting increased investment in AI solutions to optimize workflows, improve procedural accuracy, and enhance patient outcomes in interventional radiology.

**Figure 3:**
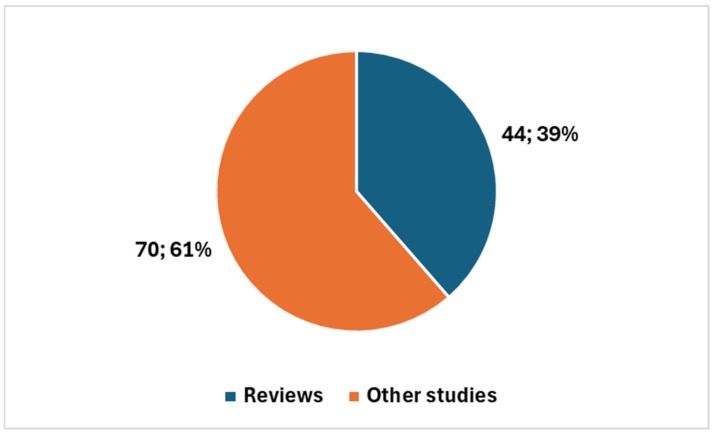
Distribution of AI-related studies in interventional radiology—comparison between review articles and primary research publications.

When analyzing the **nature and composition** of these AI-related studies, an interesting pattern emerges. Of the **114 total publications**, **44 are review articles**, while the remaining **70 studies** consist of primary research articles (Figure 3). This distribution indicates that a significant proportion of AI research in interventional radiology is dedicated to synthesizing existing knowledge, evaluating trends, and identifying future directions. Notably, the majority of these reviews are **very recent**; **42 out of 44** (**95.5%**) **have been published within the last five years**. This suggests an ongoing effort by the scientific community to assess the rapid advancements in AI and its implications for clinical practice. The predominance of recent reviews also highlights the field’s current stage of development, where researchers are still working to consolidate knowledge, establish best practices, and address key challenges such as **regulatory considerations**, **clinical validation**, **and real-world integration**.

The sharp rise in AI-related publications over the past five years can likely be attributed to several factors, which this overview aims to highlight and substantiate:

*The Acceleration of AI Research*—Advances in machine learning, deep learning, and computational power have made AI increasingly applicable to radiology.

*COVID-19 as a Catalyst*—The pandemic heightened the need for AI-driven diagnostic tools, remote assessments, and automation in medical imaging, pushing research efforts in this direction.

*Increased Funding and Interest*—Governments, healthcare institutions, and the private sector have heavily invested in AI-driven solutions for medical imaging and diagnostics.

*Regulatory and Clinical Adoption*—The increasing integration of AI into clinical workflows has driven more research to validate its effectiveness and ensure regulatory compliance.

This trend suggests that AI in interventional radiology is a rapidly expanding field, with growing recognition of its potential to enhance diagnostics, improve efficiency, and support clinical decision-making.

Box 1 **Used search keys.**

*(interventional radiology[Title/Abstract])*

*(interventional radiology[Title/Abstract]) AND ((neural network[Title/Abstract]) OR (Artificial Intelligence[Title/Abstract]) OR (deep learning[Title/Abstract]) OR (ANN[Title/Abstract]) OR (GAN[Title/Abstract]))*

*(diagnostic radiology[Title/Abstract]) AND ((neural network[Title/Abstract]) OR (Artificial Intelligence[Title/Abstract]) OR (deep learning[Title/Abstract]) OR (ANN[Title/Abstract]) OR (GAN[Title/Abstract]))*

### 3.2. The Application of IA in Interventional Radiology: A Comprehensive Overview

An analysis of the studies has been conducted, with particular focus on the integration of AI and other technologies in interventional radiology (IR), and is summarized in Table 1.

A brief complementing summary is provided below. Cornelis et al. [11] highlighted the automation and AI integration in navigation and robotic systems for percutaneous image-guided interventions, utilizing both new and established metrics to categorize and compare their capabilities. The findings indicate that none of the navigation and robotic systems have achieved full autonomy. The level of autonomy in surgical robotics (LASR) and levels of integration of advanced imaging and AI (LIAI2) scales can guide innovation by pinpointing areas for further development and integration.

Boeken et al. [12] examined the potential of AI to enhance procedural outcomes with robotics, enabling robotic systems to handle complex tasks like catheter manipulation or needle placement with greater precision and reliability. Techniques such as reinforcement learning and haptic vision are being studied to address various issues, training robots to adapt based on real-time feedback from the environment. As AI-driven robotics evolve, IR could shift towards a model where human expertise oversees the technology rather than performs the intervention itself.

Vlastaris et al. [13] explored the transformative impact of digital technologies like AI, robotics, and extended reality on IR, highlighting their potential to enhance precision, efficiency, and patient outcomes. The integration of these technologies should be considered as an aid for IR practitioners rather than as a replacement, ensuring they enhance clinical and procedural practice.

Lee et al. [14] explored the adaptation of smartphones’ built-in sensors for virtual and augmented reality uses in interventional radiology, aiming to improve accuracy and standardization for needle-based procedures like biopsy and ablation.

Barat et al. [15] explored the main research initiatives and technological advances shaping the landscape of radiology in France, including new developments in AI applications.

Matsui et al. [16] published an up-to-date review of the literature focused on the current state of AI applications in interventional oncology, including automatic segmentation of organs, tumors, and treatment areas; treatment simulation; improvement of intraprocedural image quality; prediction of treatment outcomes; and detection of post-treatment recurrence. Most AI-based methods discussed in this review are still in the research stage, with few implemented in clinical practice.

Lesaunier et al. [17] provided the medical community with the most important current and future applications of AI in IR. AI has the potential to radically change the daily practice of IR at several levels, such as multimodality management in the preoperative setting and supporting radiologists in image analysis and real-time decision-making in the perioperative setting.

Zhang et al. [18] focused on the research progress and applications of AI and robotics in interventional radiology and analyzed their potential and limitations. The findings indicated that although AI and robotics technologies are not yet widely applied in clinical settings, they are expected to significantly improve the processes and efficacy of interventional treatments.

Glielmo et al. [19] assessed the current applications and challenges of AI in IR, offering insights into decision support and outcome prediction, imaging enhancements, robotics, and touchless interactions, shaping the future of patient care.

Warran et al. [20,21] explored the current state of AI in IR in Part 1, examining its implementation across various stages: pre-procedural, intra-procedural, and post-procedural. In Part 2, they outlined a hierarchy of potential risks and harms associated with AI and provided a checklist to guide the safe deployment of AI technologies in clinical IR settings.

Geevarghese et al. [22] explored recent developments within interventional oncology, highlighting its potential impact facilitated by AI, personalized medicine, and imaging innovations. The integration of AI in interventional oncology promises to accelerate tumor detection and characterization, guide treatment strategies, and refine predictive models.

Campbell 4th et al. [23] summarized the design principles of ChatGPT relevant to healthcare and highlighted tasks and activities with the greatest potential for ChatGPT utilization in the practice of IR. These tasks involve patient-directed and physician-directed communications to convey medical information efficiently and act as a medical decision support tool.

O’Brien et al. [24] examined the literature to contextualize how AI is currently being implemented in interventional radiology from a preoperative, intraoperative, and postoperative perspective.

Gaddum and Chapiro [25] provided a guide for interventional radiologists to critically appraise AI research and products by identifying 12 key considerations: AI’s relevance to the clinical problem, type of AI algorithm used, data quality and annotation, accuracy reporting, standardized reporting applicability, reproducibility and transparency, algorithm validation, interpretability, impact on IR, pathway to clinical translation, clinical benefit and cost-effectiveness, and regulatory framework.

Lanza et al. [26] provided a brief overview of the current status and potential role of robotics in IR. The technical developments in robotics and navigational systems using computed tomography image guidance, magnetic resonance image guidance, and ultrasound image guidance were analyzed.

Von Ende et al. [27] described the current and possible future applications of AI, radiogenomics, and augmented and virtual reality in IR, also describing the challenges and limitations that must be addressed before these applications can be fully implemented into common clinical practice.

Posa et al. [28] provided a comprehensive overview of the most valuable, well-established, and promising AI and digital health innovations, aimed at empowering physicians to incorporate these technologies into clinical practice. Their work particularly emphasized advancements in the field of interventional oncology, encouraging greater physician engagement with these cutting-edge tools.

Boeken et al. [29] provided an update on the current status of radiomics and AI research, analyzed upcoming challenges, and also discussed the main applications of AI in IR to help radiologists better understand and critique articles reporting AI in medical imaging.

Kallini et al. [30] explored the evolution of AI from its theoretical foundations in the 1950s to modern advancements. While AI made significant progress in radiology, its application in IR remained in its early stages. This article summarized AI’s role in radiology and IR, highlighting past advancements and future potential.

Waller et al. [31] examined how the implementation of AI in diagnostic radiology and IR can improve image analysis, aid in diagnosis, and suggested appropriate interventions, clinical predictive modeling, and trainee education.

Seah et al. [32] provided an overview of machine learning, radiomics, and AI in the field of IR, enumerating the possible applications of such techniques, also describing techniques to overcome the challenge of limited data when applying these techniques in IR.

Mazaheri et al. [33] examined a few challenges contributing to this scarcity of AI applications in IR, including inherent specialty challenges, regulatory hurdles, intellectual property, raising capital, and ethics.

Desai et al. [34] described AI technologies that have been developed within the IR suite, as well as some future work with a focus on AI’s potential impact in pediatric interventional medicine.

D’amore et al. [35] highlighted the current role of machine learning and AI in the field of interventional oncology. Software development can increase the detection of tumors through routine screening and improve diagnostic accuracy in classifying tumors.

Gurgitano et al. [36] discussed the potential of AI applications in IR, which range beyond computer vision and diagnosis, to include screening and modeling of patient selection, predictive tools for treatment planning and navigation, and training tools.

Iezzi et al. [37] integrated evidence-reported literature and experience-based perceptions to assist not only residents and fellows who are training in IR but also practicing colleagues who are approaching locoregional mini-invasive treatments.

Table 1 presents a summary of the analyzed studies with their focus and the emerging role of AI (and any other technologies).

**Table 1:** Summary of the analyzed studies with their focus and the emerging role of AI (and any other technologies).

Study	Brief Description	Focus	AI and Technology Integration
[11] Evaluation of navigation and robotic systems for percutaneous image-guided interventions: A novel metric for advanced imaging and artificial intelligence integration	This study reviews clinically validated robotic and navigation systems for percutaneous image-guided interventions, focusing on integrating AI and advanced imaging. It introduces a novel metric, LIAI2, for assessing AI and imaging integration.	The paper evaluates the extent of AI and advanced imaging integration in robotic systems for percutaneous procedures, aiming to enhance automation levels and precision.	The integration of AI aims to improve procedural accuracy and system autonomy, helping with tasks like needle placement and biopsies. This paper introduces a novel taxonomy to measure AI and imaging integration, addressing current limitations and driving future innovation in autonomous navigation.
[12] The Role and Future of Artificial Intelligence in Robotic Image-Guided Interventions	This review explores AI’s transformative potential in robotic-assisted interventional radiology (IR). It discusses AI’s role in handling tasks like catheter manipulation and needle placement, as well as current challenges such as limited decision-making capabilities.	The study focuses on how AI can improve robotic systems’ precision, efficiency, and reliability in IR interventions, enabling automation in complex procedures.	AI integration in robotic systems enhances precision and adaptability, particularly through reinforcement learning. While AI-driven robotics are expected to take over more tasks, human oversight remains essential to ensure safety and control during complex procedures.
[13] The Transformative Impact of AI, Extended Reality, and Robotics in Interventional Radiology: Current Trends and Applications	This article explores the integration of AI, robotics, and extended reality (XR) in interventional radiology, showcasing how these technologies are reshaping procedures and improving outcomes. It discusses the challenges of standardization across healthcare settings.	The paper emphasizes the synergy between AI, XR, and robotics to enhance image-guided interventions’ precision and efficiency in IR.	AI, robotics, and XR are being integrated to enhance diagnostic accuracy and procedural precision. AI is improving outcomes, while XR aids in visualizing complex anatomy, and robotics reduces human error. These technologies complement clinicians’ skills and aim to optimize patient care without replacing human expertise.
[14] Smartphone Technology for Applications in Image-Guided Minimally Invasive Interventional Procedures	This study investigates the use of smartphone technologies, such as augmented reality (AR), in image-guided minimally invasive procedures like biopsies and ablations. It explores how smartphones could enhance the accuracy and efficiency of needle-based interventions.	The focus is on the use of smartphones and AR to improve minimally invasive IR procedures by offering portable, real-time image guidance.	The integration of AR with smartphone sensors allows for real-time visualization of the procedural environment, offering greater precision in navigating complex anatomy. However, further research is needed to refine the technology and optimize its clinical use in IR.
[15] Imaging in France: 2024 Update	This article reviews recent advances in diagnostic and interventional radiology in France, particularly the integration of AI into diagnostic imaging. It highlights France’s contributions to improving diagnostic accuracy and advancing minimally invasive treatments in IR.	The paper focuses on technological advancements in AI-driven diagnostic imaging and their impact on IR, emphasizing innovations in tumor detection and advanced image fusion techniques.	AI has become central to improving diagnostic imaging in France, with applications in tumor detection, CT imaging, and image fusion. These advancements are enhancing the accuracy of diagnoses and treatment planning in IR. The study also discusses AI’s role in improving workflows and patient outcomes in interventional procedures.
[16] Applications of artificial intelligence in interventional oncology: An up-to-date review of the literature	This comprehensive review examines the role of AI in interventional oncology, particularly in image-guided therapies such as tumor embolization and ablation. It discusses AI tasks like tumor segmentation, outcome prediction, and recurrence detection.	The study focuses on how AI is revolutionizing interventional oncology through automation and enhanced treatment prediction.	AI in interventional oncology is being used to automate segmentation, predict outcomes, and detect recurrence after treatments. The integration of deep learning and radiomics is crucial in these tasks, improving treatment precision and patient management. More research is needed to validate these AI applications in clinical settings.
[17] Artificial intelligence in interventional radiology: Current concepts and future trends	This article explores the state of AI in interventional radiology, emphasizing its applications in preoperative, perioperative, and postoperative care. It highlights AI’s role in patient selection and outcome prediction.	The paper highlights both current and potential future applications of AI in IR, including image analysis, patient management, and real-time decision-making.	AI is enhancing the planning and execution of IR procedures, particularly in managing multimodal data and predicting patient outcomes. AI’s ability to support decision-making and improve robotic system capabilities is transforming procedural approaches and increasing procedural autonomy.
[18] How AI and Robotics Will Advance Interventional Radiology: Narrative Review and Future Perspectives	This review discusses the convergence of AI and robotics in advancing interventional radiology, particularly focusing on deep learning, machine learning, and convolutional neural networks (CNNs). It examines how AI and robotics can enhance precision across different specialties in IR.	The focus is on AI and robotics’ integration to improve procedural accuracy in IR, particularly in specialties like oncology, neurology, and cardiology.	AI and robotics are improving procedural precision in IR, with deep learning and CNNs automating complex tasks and predicting patient outcomes. The review discusses the potential for AI-driven robotics in IR, highlighting challenges such as integration and the need for standardization.
[19] Artificial intelligence in interventional radiology: state of the art	This paper provides an overview of AI applications in interventional radiology, emphasizing how AI supports decision-making, enhances imaging quality, and improves robotic interactions. It also addresses challenges to AI adoption, such as the complexity of IR procedures and the lack of standardization.	The focus is on AI’s role in enhancing decision support, imaging, and procedural execution in IR, with a focus on its transformative potential.	AI is significantly contributing to IR by improving decision support systems, enhancing imaging technologies like fusion imaging, and enabling robotic systems to function with greater precision. AI’s role in touchless interaction and virtual biopsies is also explored, though adoption is hindered by variability in procedural practices across institutions.
[20] An Introductory Guide to Artificial Intelligence in Interventional Radiology: Part 2: Implementation Considerations and Harms	This article provides a comprehensive look at the risks and challenges of implementing AI in interventional radiology (IR). It discusses potential harms associated with AI deployment, including overreliance on automated systems, and offers a practical checklist for clinicians to safely integrate AI technologies into clinical practice.	The article focuses on the practical considerations necessary for AI integration in IR. It emphasizes understanding and managing the risks involved in deploying AI systems, ensuring clinicians are equipped with strategies for safe implementation. It discusses regulatory oversight and monitoring mechanisms to prevent adverse effects on patient care.	The study presents AI as a tool with significant benefits but also highlights the importance of evaluating AI technologies based on their risk potential. It stresses the need for proper risk assessment and regulatory frameworks to ensure patient safety during AI deployment in clinical settings.
[21] An Introductory Guide to Artificial Intelligence in Interventional Radiology: Part 1 Foundational Knowledge	This foundational guide introduces interventional radiologists to the core concepts of AI. It outlines how AI is applied across different procedural stages, including pre-procedural planning, intra-procedural guidance, and post-procedural monitoring, aiming to enhance diagnostic accuracy and workflow efficiency.	The focus of this article is on educating clinicians about the role of AI in IR, explaining how AI can be used to optimize clinical workflows and decision-making at various stages of procedures. It also introduces a classification system for AI models to help clinicians understand the complexity and suitability of different AI technologies for their practice.	AI is framed as an essential tool for improving clinical outcomes and procedural efficiency in IR. The guide emphasizes the importance of AI across all procedural stages, helping radiologists understand how AI technologies can be effectively integrated into their daily practice.
[22] Interventional Oncology: 2024 Update	This review examines the integration of AI in interventional oncology, focusing on its role in imaging, treatment planning, and patient care. It explores AI’s potential to enhance tumor detection and personalize treatments, ultimately aiming to improve patient outcomes in oncology.	The focus is on AI’s impact on interventional oncology, particularly in improving diagnostic accuracy and tailoring treatment strategies to individual patients. The article also discusses the evolving training requirements for clinicians to handle AI technologies in oncology and their growing importance in clinical decision-making.	AI is integrated with advanced imaging technologies to improve tumor detection and characterization. It also plays a key role in personalizing treatment plans based on genomic data, offering a more individualized approach to oncology care.
[23] Understanding ChatGPT for Evidence-Based Utilization in Interventional Radiology	This study explores the use of ChatGPT, a language-based AI model, to support evidence-based decision-making in interventional radiology. It discusses how this AI tool can assist with patient communication, data interpretation, and clinical decision support.	The focus is on ChatGPT’s potential to enhance communication between clinicians and patients, as well as its ability to assist in decision-making processes by providing evidence-based insights and recommendations.	ChatGPT is presented as a valuable tool for streamlining communication in IR, helping clinicians access information quickly and support clinical decision-making by interpreting data and providing relevant recommendations.
[24] Current Applications of Algorithmic Artificial Intelligence in Interventional Radiology: A Review of the Literature	This review provides an overview of the current uses of algorithmic AI in interventional radiology, exploring its applications across various procedural stages. It discusses how AI is used to optimize imaging, assist in decision-making, and predict procedural outcomes.	The focus is on the broad applications of AI in IR, detailing its use in improving imaging quality, selecting patients for procedures, and predicting treatment outcomes. It emphasizes the potential of AI to streamline workflows and enhance the precision of interventions.	AI is used to enhance imaging capabilities, improve diagnostic accuracy, and assist with procedural decision-making. The review highlights AI’s growing role in predicting outcomes, reducing errors, and increasing efficiency in IR procedures.
[25] An Interventional Radiologist’s Primer of Critical Appraisal of Artificial Intelligence Research	This primer guides interventional radiologists in evaluating AI research, providing criteria for assessing the accuracy, reliability, and clinical applicability of AI models. It helps clinicians critically analyze AI technologies to ensure they meet clinical needs.	The focus is on equipping clinicians with the skills to critically appraise AI research and products. It provides a structured approach to evaluating AI models, focusing on accuracy, data quality, and the relevance of AI applications in clinical practice.	The article highlights the importance of critically assessing AI tools before integrating them into clinical practice. It ensures that clinicians can evaluate AI models based on scientific rigor and clinical relevance, promoting informed decisions on technology adoption.
[26] Robotics in Interventional Radiology: Review of Current and Future Applications	This review explores the current and future applications of robotics in IR, particularly in procedures involving image-guided navigation. It examines how AI-powered robotic systems are improving the precision and safety of minimally invasive interventions.	The focus is on the integration of robotic systems in IR, with an emphasis on AI’s role in enhancing procedural accuracy. It discusses the potential for robotic systems to transform IR by improving precision, reducing human error, and facilitating complex procedures.	Robotics, combined with AI, is transforming IR by enhancing precision and safety in interventions. AI-driven systems assist in tasks like navigation and decision-making, particularly in minimally invasive procedures, leading to better outcomes and reduced procedural risks.
[27] Artificial Intelligence, Augmented Reality, and Virtual Reality Advances and Applications in Interventional Radiology	This review examines how AI, augmented reality (AR), and virtual reality (VR) are converging to enhance interventional radiology. It highlights the potential of these technologies to improve diagnostics, guide treatment planning, and assist in complex procedures.	The focus is on the intersection of AI with AR and VR, exploring how these technologies can work together to provide clinicians with enhanced visualizations, decision support, and real-time feedback during procedures.	AI, AR, and VR are integrated to provide a more immersive, accurate, and interactive approach to diagnostics and treatment planning in IR. These technologies are improving procedural outcomes by offering real-time insights and visual assistance to clinicians.
[28] Technological Advancements in Interventional Oncology	This article explores the transformative impact of artificial intelligence in interventional oncology, focusing on the integration of AI for the analysis of large datasets and its use in predictive modeling for treatment response. It discusses the evolution of AI applications in diagnosing and treating cancers, highlighting AI’s ability to improve precision in tumor detection and treatment planning. The review provides insights into how AI and digital health technologies are enhancing workflows in clinical settings, particularly within interventional oncology.	The paper highlights AI’s role in improving diagnostic precision in oncology by analyzing vast medical data, aiding in tumor detection, and optimizing treatment planning. It discusses AI’s potential to predict treatment responses and guide clinical decisions, transforming the approach to personalized cancer care and workflow efficiency in oncology interventions.	AI has proven transformative in interventional oncology, enhancing tumor detection and treatment precision. By leveraging data and predictive modeling, AI is improving diagnostic accuracy and clinical decision-making, although challenges remain regarding data quality and clinical adoption.
[29] Artificial intelligence in diagnostic and interventional radiology: Where are we now?	This article examines the current state of AI in radiology, specifically in diagnostic and interventional radiology. It explores how AI, especially in conjunction with radiomics, is revolutionizing imaging processes in both diagnostics and interventions. The paper emphasizes how AI technologies are increasingly being used to assist radiologists by providing enhanced image analysis, automating repetitive tasks, and supporting clinical decision-making. Interventional radiology is poised to benefit from AI’s ability to enhance procedural guidance, patient selection, and real-time monitoring.	The article discusses the integration of AI in improving image analysis and automating tasks, making diagnostic processes more efficient. It also explores AI’s growing role in interventional radiology, including procedural guidance, real-time decision support, and improving patient outcomes.	AI is improving efficiency in radiology by automating tasks and enhancing image analysis. In interventional radiology, AI is increasingly used for procedural guidance, real-time monitoring, and decision-making, though challenges like data quality and integration persist.
[30] Artificial Intelligence in Interventional Radiology	This article reviews the historical development of artificial intelligence in medicine, focusing on its applications in interventional radiology. It traces the evolution of AI from its early conceptual stages to its current role in enhancing diagnostic capabilities and procedural guidance. AI in interventional radiology is mainly being used to assist in tumor detection, automate routine tasks, and improve procedural outcomes. The article also discusses the potential future impact of AI in revolutionizing interventional radiology by enhancing workflow and diagnostic precision.	The paper outlines the historical evolution and current applications of AI in interventional radiology, focusing on its role in tumor detection, automation of routine tasks, and improving procedural outcomes. It also looks at the future potential for AI to further enhance diagnostic precision and workflow efficiency.	AI is significantly enhancing tumor detection, procedural guidance, and workflow automation in interventional radiology. The paper emphasizes the future potential of AI, particularly in supporting real-time decision-making and optimizing clinical outcomes.
[31] Applications and challenges of artificial intelligence in diagnostic and interventional radiology	This literature review discusses the implementation of machine learning (ML) and deep learning (DL) techniques in radiology, including their applications in interventional radiology. The paper explores how AI can enhance image analysis, support diagnosis, assist in predicting management decisions, and improve clinical outcomes. It also addresses the challenges of implementing AI in clinical settings, such as the need for accurate, representative datasets and ethical considerations.	This review focuses on the applications of machine learning and deep learning in radiology, especially in interventional radiology. It highlights AI’s potential to improve image analysis, diagnostic accuracy, and patient management decisions, while also addressing the challenges posed by data quality and ethical issues in clinical adoption.	AI, particularly machine learning and deep learning, is revolutionizing diagnostic and interventional radiology by improving image analysis and supporting clinical decisions. However, challenges like data quality, training requirements, and ethical concerns hinder widespread implementation.
[32] Prime Time for Artificial Intelligence in Interventional Radiology	This article highlights the growing interest in AI within interventional radiology, focusing on the integration of machine learning and radiomics. It examines the potential applications of AI to enhance procedural planning, image-guided interventions, and patient monitoring. The paper emphasizes the readiness of interventional radiology to lead AI development, given its standardized data formats and data-rich environment. The article also discusses the need for research into overcoming challenges related to limited datasets and the integration of AI into clinical workflows.	The paper discusses the promising applications of AI in procedural planning, image-guided interventions, and patient monitoring within interventional radiology. It highlights the sector’s data-rich environment and standardization as factors that could help accelerate AI adoption, though challenges such as limited datasets remain.	AI has the potential to revolutionize procedural planning and patient monitoring in interventional radiology. However, the paper stresses that overcoming challenges like limited datasets and integrating AI into clinical practice are key hurdles that need addressing.
[33] Challenges of Implementing Artificial Intelligence in Interventional Radiology	This paper discusses the slow pace of AI adoption in interventional radiology compared to diagnostic radiology, identifying key challenges such as regulatory hurdles, data limitations, and ethical concerns. Despite AI’s potential to transform interventional radiology by enhancing procedural efficiency and diagnostic accuracy, the article highlights how these barriers have delayed widespread implementation. It calls for continued engagement with stakeholders to define clinically relevant use cases and prioritize resources effectively.	The article examines the slow adoption of AI in interventional radiology, emphasizing the regulatory and data challenges, as well as ethical concerns. It calls for collaboration among stakeholders to address these barriers and unlock the full potential of AI in this field.	The article identifies key barriers to AI adoption in interventional radiology, including regulatory hurdles, data quality, and ethical concerns. It stresses the need for collaboration to address these challenges and expedite AI integration into clinical practice.
[34] Current and emerging artificial intelligence applications for pediatric interventional radiology	This review examines the potential applications of AI in pediatric interventional radiology, an area that remains underserved in terms of AI technologies. The article discusses how AI can be utilized to improve image analysis, assist in diagnosis, and enhance procedural guidance in pediatric patients. Given the unique challenges of treating children, the paper emphasizes the importance of developing AI tools tailored to the specific needs of pediatric patients.	The article explores how AI can improve diagnostic and procedural outcomes in pediatric interventional radiology. It stresses the need for AI tools tailored to the unique anatomical and treatment needs of pediatric patients.	AI in pediatric interventional radiology holds promise for improving image analysis and procedural guidance, but the article highlights the need for AI tools specifically designed to meet the unique challenges of treating children.
[35] Role of Machine Learning and Artificial Intelligence in Interventional Oncology	This review highlights the role of AI and machine learning in interventional oncology, emphasizing their potential to enhance image analysis, intraprocedural guidance, and diagnostic accuracy.	The study explores how AI improves cancer detection, tumor classification, and treatment selection by predicting outcomes based on clinical and radiologic data. It also discusses challenges such as data processing, model validation, workflow integration, and ethical considerations.	AI and ML contribute to procedural guidance with precise needle tracking and image fusion, minimizing damage to healthy tissue while optimizing tumor treatment.
[36] Interventional Radiology ex-machina: Impact of Artificial Intelligence on Practice	This article examines the role of AI in interventional radiology, focusing on its potential to improve minimally invasive treatments and procedural accuracy.	The study explores AI applications in screening, patient selection modeling, predictive treatment planning, and procedural navigation. It also discusses the impact of augmented reality, mixed reality, and virtual reality in enhancing interventional radiology workflows.	AI, ML, and deep learning improve image analysis, procedural precision, and decision-making. Augmented and virtual reality technologies assist in enhancing interventional radiology procedures.
[37] Artificial Intelligence in Interventional Radiology: A Literature Review and Future Perspectives	This literature review discusses AI’s impact on interventional radiology, integrating evidence-based insights and expert perspectives to highlight current applications and future directions.	The study focuses on AI’s role in lesion detection, segmentation, procedural planning, and interventional oncology. It examines AI’s ability to provide prognostic insights and enhance minimally invasive treatments.	AI-driven computational algorithms, machine learning, and artificial neural networks improve diagnostic accuracy, procedural workflows, and personalized treatment planning in interventional radiology.

The integration of AI into interventional radiology IR is rapidly transforming various procedural and operational aspects of the field. AI-driven technologies are enhancing precision, optimizing workflows, and improving patient outcomes across multiple domains of IR. Table 2 provides an overview of key AI applications in IR, categorized by their specific contributions to robotic navigation, oncology interventions, predictive decision support, minimally invasive procedures, image fusion, workflow efficiency, real-time monitoring, and ethical and regulatory considerations.

AI plays a crucial role in robotic and navigation systems, where it enhances the accuracy of complex procedures such as needle insertion, biopsy guidance, and catheter placement ([11,12,18,19]). By analyzing imaging data in real time, AI helps clinicians determine the most precise trajectory for interventions, minimizing risks and improving procedural success. Similarly, AI-driven technologies in oncology interventions contribute to tumor detection, ablation, and post-treatment monitoring, assisting in precise tumor localization and improving follow-up care ([16,20,28,31,32,35]).

Another significant application of AI in IR lies in predictive decision support, where it facilitates outcome prediction and personalized treatment strategies ([17,19,23,33,37]). By analyzing large datasets, AI helps anticipate complications and optimize treatment plans, enhancing clinical decision-making. AI also supports minimally invasive procedures, improving access point localization, biopsy precision, and ablation targeting, which leads to better procedural accuracy and reduced patient recovery times ([13,14,15,25,34,35]).

In the realm of imaging, AI significantly improves diagnostic accuracy by integrating and enhancing imaging modalities such as CT, MRI, and ultrasound ([11,19,22,26,27,35]). By fusing multiple sources of imaging data, AI provides clinicians with a more comprehensive and precise visualization, aiding in complex diagnoses and treatment planning. Additionally, AI optimizes workflow efficiency by automating routine tasks, scheduling, and streamlining clinical processes, thereby reducing administrative burdens and allowing physicians to focus on patient care ([21,25,29,36]).

The table below summarizes these key AI applications, highlighting the range of medical procedures they impact and the specific advancements they bring to interventional radiology. The contributions of the reviews are often transversal across categories; however, efforts have been made to identify the dominant areas within each category.

**Table 2:** Categorization of the studies.

Category	Study Range	Medical Applications	Expanded Medical Applications Description
AI in Robotic and Navigation Systems	[11,12,18,19]	Needle Insertion, Biopsy Guidance, Catheter Placement	AI integration in robotic and navigation systems enhances the precision of complex procedures like needle insertion, biopsy guidance, and catheter placement. By analyzing imaging data in real-time, AI systems help identify the most accurate path for needle insertions and biopsies, minimizing damage to healthy tissue and improving the precision of sampling. In catheter placement, AI-driven systems provide constant adjustments to ensure accurate targeting, improving both procedural outcomes and patient safety. These AI-enhanced robotic systems reduce human error, improve targeting, and optimize real-time decision-making, resulting in fewer complications and faster recovery for patients.
AI in Oncology Interventions	[16,20,28,31,32,35]	Tumor Ablation, Tumor Detection, Post-Treatment Monitoring	In oncology, AI contributes to improved precision in tumor treatments such as ablation and embolization. By providing advanced image processing and automatic segmentation, AI enhances the accuracy of targeting tumors while minimizing damage to surrounding healthy tissue. AI systems also aid in early tumor detection by analyzing various imaging modalities and highlighting potential malignancies. Additionally, AI tools monitor patients post treatment, detecting early signs of tumor recurrence, predicting patient outcomes, and assessing the effectiveness of treatments, thus enabling more personalized follow-up care.
AI in Predictive Decision Support	[17,19,23,33,37]	Outcome Prediction, Personalized Treatment, Decision-Making Support	AI in predictive decision support systems analyzes patient data to predict outcomes, suggest personalized treatments, and assist in real-time clinical decisions. This system provides clinicians with a more precise understanding of potential risks and complications, improving diagnostic accuracy and patient safety. By continuously learning from evolving patient data, AI supports clinicians in making data-driven decisions that adapt to individual patient needs. The predictive capability of AI optimizes treatment plans and allows clinicians to anticipate complications, ensuring the most effective interventions are chosen.
AI in Minimally Invasive Procedures	[13,14,15,25,34,35]	Biopsy, Ablation, Access Point Localization	AI significantly improves the precision and safety of minimally invasive procedures by guiding access point localization, enhancing biopsy accuracy, and improving ablation targeting. Real-time AI-driven imaging systems provide immediate feedback, helping clinicians navigate anatomical structures with greater precision, thereby reducing the risk of complications. AI-assisted procedures like biopsies and ablations benefit from increased targeting accuracy, minimizing damage to healthy tissues and leading to faster patient recovery. Additionally, AI technologies optimize pre-procedural planning, allowing for more efficient execution of interventions.
AI in Image Fusion and Enhancement	[11,19,22,26,27,35]	Tumor Detection, Image Integration, Imaging Precision	AI-powered image fusion and enhancement techniques integrate multiple imaging modalities (e.g., CT, MRI, ultrasound) to provide clinicians with a more comprehensive view of a patient’s condition. This integration enhances diagnostic accuracy, especially for complex cases such as tumor detection, vascular anomalies, and surgical planning. By merging different imaging sources, AI helps to create more precise, detailed images, enabling more informed decision-making. This fusion is particularly valuable when dealing with difficult-to-diagnose conditions, as it provides a clearer picture of the patient’s anatomy, guiding clinicians to the most accurate diagnosis and treatment plan.
AI in Workflow and Efficiency Optimization	[21,25,29,36]	Task Automation, Scheduling, Process Streamlining	AI improves the operational efficiency of interventional radiology by automating routine tasks such as scheduling, data entry, and image processing. This allows healthcare providers to focus more on patient care rather than administrative tasks. AI algorithms can also streamline procedural planning by analyzing patient data and procedural variables to select the most efficient approach, optimizing resource allocation, reducing unnecessary interventions, and ensuring timely patient care. Additionally, AI helps predict patient volume and plan staffing, ensuring better resource utilization and smoother workflow in clinical settings.

### 3.3. Emerging Opportunities and Challenges

The integration of artificial intelligence (AI) into interventional radiology (IR) promises to revolutionize the field by enhancing various aspects of clinical practice. However, the successful adoption and implementation of AI technologies face several opportunities and challenges, as identified in the studies reviewed. Figure 3 reports a sketch of these opportunities and challenges.

**Opportunities in the Application of AI in IR**

**Figure 4:**
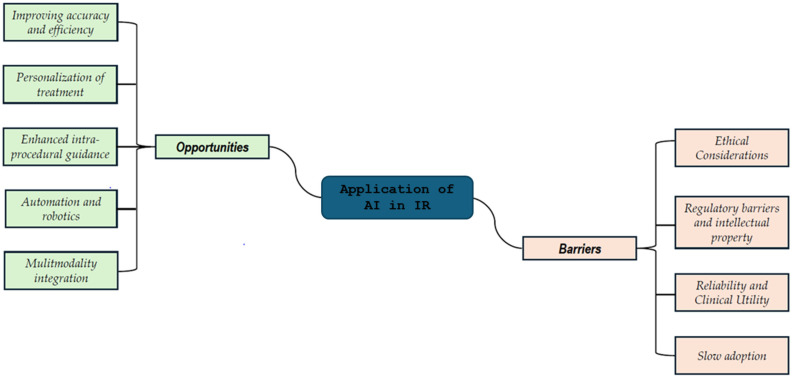
A graphical summary of the opportunities and challenges associated with the application of AI in IR.

As reported in Figure 4, the opportunities are as follows:

Improving Accuracy and Efficiency: One of the most significant opportunities provided by AI in IR is its ability to improve both accuracy and efficiency during procedures. AI systems can assist healthcare professionals by analyzing medical images with remarkable precision, leading to faster diagnosis and more accurate treatment decisions. Studies have shown that AI-based tools can streamline clinical workflows and enhance procedural efficiency by automating tasks [13,17,29]. AI-guided robotic systems can further reduce human error and variability between clinicians, ensuring more consistent outcomes in interventions such as biopsies, ablations, or embolizations. Additionally, AI can speed up diagnosis by suggesting personalized interventions, thus minimizing procedural time and improving patient throughput [34].

*Personalization of Treatment*: The application of AI in IR offers the possibility of highly personalized treatment strategies. Through machine learning algorithms, AI can process vast amounts of clinical and radiological data to predict the likely outcomes of various interventions, enabling clinicians to select the most appropriate treatment for each patient [17,22,26]. This data-driven approach not only improves the effectiveness of the chosen interventions but also helps in minimizing side effects, optimizing patient outcomes, and reducing the burden on healthcare resources [35]. Personalized treatment planning is particularly useful in oncology, where AI can assist in identifying the most effective tumor treatment options based on the specific characteristics of each case.

*Enhanced Intra-Procedure Guidance*: AI-driven technologies are transforming intra-procedural guidance by providing real-time support to clinicians. Advanced imaging techniques such as needle tracking and real-time image fusion, powered by AI, enable highly targeted interventions that minimize damage to healthy tissues and enhance the effectiveness of treatments like tumor ablation or embolization [22,35]. Furthermore, emerging AI tools like reinforcement learning and haptic vision are enabling robotic systems to adapt in real-time to feedback from the environment, significantly improving the precision of procedures [12]. These technologies ensure that interventions are both safer and more efficient, contributing to better patient outcomes.

*Automation and Robotics*: AI is revolutionizing the role of robotics in IR by allowing for greater automation of complex procedures. Robotic systems that are controlled and guided by AI can perform tasks with a higher degree of accuracy and reliability compared to human hands alone [11,12,17,19]. This includes not only assisting in navigation during image-guided interventions but also potentially performing fully autonomous tasks. The introduction of autonomous systems could redefine the role of the interventional radiologist, shifting from being the primary operator to overseeing and monitoring the robotic technology [26]. Such advancements could lead to the development of fully automated procedures, reducing human intervention and error while improving procedural outcomes.

*Multimodality Integration*: AI’s capacity to integrate data from multiple imaging modalities (such as CT, MRI, ultrasound, and more) is one of its most promising features in IR. The ability to combine various types of imaging data allows for a more comprehensive understanding of a patient’s condition and facilitates better-informed decision-making. AI can synthesize these data to create more accurate models for patient selection, prediction of outcomes, and planning of treatments [17,26,29] This multimodal integration enhances the accuracy of diagnosis and treatment planning, particularly in complex cases where a single imaging modality may not provide sufficient information.

**Barriers to AI Integration in IR**

Despite the tremendous opportunities AI offers in IR, several barriers remain that need to be addressed to ensure its widespread adoption and effective implementation *as reported in* Figure 4.

Ethical Considerations: The introduction of AI into IR raises significant ethical concerns. One major challenge is the lack of transparency in AI decision-making processes. Many AI systems operate as “black boxes”, meaning that the logic behind their recommendations is not always fully understood by clinicians. This lack of transparency can undermine trust in AI-driven decisions, especially when these systems influence patient outcomes. Additionally, ethical concerns regarding data privacy and the use of patient information persist, as AI systems require large, accurately labeled datasets to function effectively [27,31,33,35]. Ensuring that these datasets are representative and unbiased is crucial to avoid perpetuating health disparities.

*Regulatory Barriers and Intellectual Property*: Given the issues that emerge as AI technologies continue to evolve, regulatory frameworks must keep pace to ensure patient safety and system effectiveness. In many countries, the regulation of AI-driven medical devices is still developing, creating uncertainty about the approval and implementation of these systems in clinical practice. The need for rigorous evaluation and classification of AI technologies based on risk level is essential to ensure that AI tools meet safety standards before being deployed in the clinical setting [25,33]. Furthermore, intellectual property (IP) concerns surrounding AI algorithms and the data they use can complicate the development and deployment of AI systems in healthcare. Conflicts over the ownership of AI technologies and the data they generate could delay the integration of these innovations in IR.

*Reliability and Clinical Utility*: Many AI-based systems are still in the research phase and have not yet been fully validated for clinical use. Although AI has demonstrated promising results in controlled studies, the consistency and reliability of these systems in real-world clinical settings remain uncertain. Further research is needed to assess the clinical utility and long-term benefits of AI technologies in IR. Until more extensive clinical trials are conducted and these systems prove their reliability, widespread adoption may be hindered [16,19,31]. Clinicians need to be confident in the reliability of AI tools before incorporating them into routine practice.

*Slow Adoption*: The pace of AI adoption in IR is slower compared to diagnostic radiology, where AI technologies have already been more widely accepted and integrated into clinical workflows. The integration of AI in IR faces additional complexities, such as the need for specialized training, changes to clinical workflows, and the integration of AI with existing technologies. Furthermore, concerns about the cost and the need for extensive validation studies have slowed down the widespread use of AI in interventional procedures [17,19,27,33]. While AI is making strides in diagnostic radiology, it is only beginning to establish itself in IR, and a more gradual adoption process is expected.

Overall, the integration of AI into interventional radiology offers significant opportunities to improve accuracy, efficiency, and patient outcomes. However, challenges such as ethical considerations, regulatory hurdles, and concerns regarding the reliability and clinical utility of AI systems must be addressed to ensure their successful implementation. By overcoming these barriers and continuing to advance AI technologies, the future of IR holds immense potential for transforming patient care and procedural efficiency. The figure accompanying this discussion outlines the primary opportunities and barriers identified in the studies reviewed.

## 4. Discussion

The discussion is structured into five sections, each aimed at providing a comprehensive analysis and interpretation of the study’s findings:

*Section 4.1*: This section reports a synoptic of the organization of the discussion detailing the rationale based on the output of the results.

*Section 4.2*: This section presents the key evidence emerging from the overview, outlining the added values of this research. This section establishes the foundation for understanding the contributions of the study and situates them within the broader context of the field.

*Section 4.3*: This section delves into the positioning of the study within the broader and more comprehensive field of radiology. It aims to contextualize the research by highlighting how it contributes to, and interacts with, the ongoing advancements and trends in the radiology sector.

*Section 4.4*: As a natural progression from the emerging opportunities and barriers outlined earlier in the NRR, this section examines trends within recent cutting-edge primary studies. It assesses their contributions and evaluates how these groundbreaking works align with the broader themes and insights identified in the review.

*Section 4.5*: This section focuses on the global positioning of various organizations and regulatory bodies in terms of their guidelines and recommendations. It examines how international institutions and authorities influence the practice of radiology and interventional procedures, providing a perspective on how standardization, ethical considerations, and best practices in the field are faced.

Finally, Section 4.6 identifies the boundaries of the overview and reports the limitations.

This structured approach ensures a detailed and holistic discussion, providing both a synthesis of findings and a forward-looking perspective that connects the study’s insights with practical implications and future research directions. Each section is designed to build on the previous one, creating a cohesive narrative that reflects the complexity and potential of the field.

### 4.1. Synoptic Diagram

**Figure 5:**
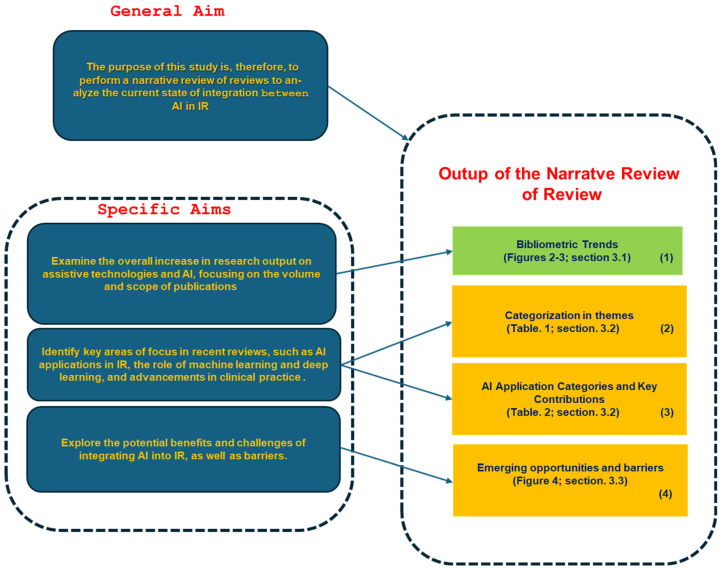
Synoptic diagram of the study structure: from bibliometric trends to AI application categorization and comparative analysis.

**Figure 6:**
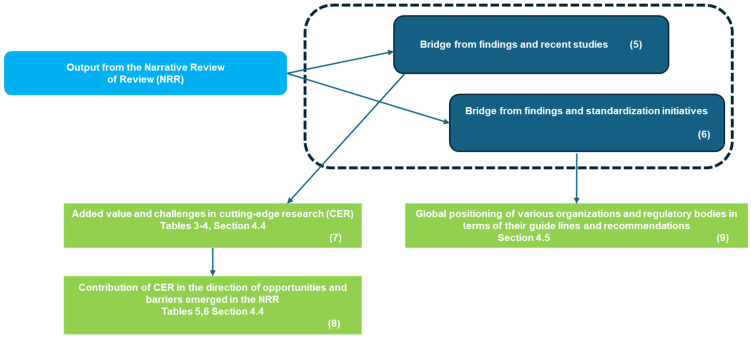
Logical progression from NRR findings to the CER contribution and the international path to standardization.

Figure 5 and Figure 6 present two synoptic diagrams that outline the rationale behind the design of the **narrative review of reviews** (**NRR**). These diagrams provide a structured visual representation of how the study was developed, showing the logical sequence of its different phases and how they interconnect.

**First Diagram (Figure 5): Linking Objectives to Analysis**

The first diagram (Figure 5) illustrates how the study was structured based on its **general objective** and **three specific objectives**. The logical progression follows a top-down approach:

- **Block 1**: This block represents the **bibliometric trends** reported in Figure 2 and Figure 3 (Section 3.1). These trends were analyzed to provide an overview of the scientific production on AI applications in IR, forming the foundation for the subsequent steps of the study.
- **Block 2**: This block corresponds to the **categorization of studies by thematic areas**, as presented in Table 1 (Section 3.2). This categorization allowed for the organization of the reviewed studies according to key themes, facilitating a structured comparison.
- **Block 3**: Building upon the thematic categorization, this block highlights the **comparative side-by-side analysis** of the studies. The classification of AI applications was refined, as reported in Table 2 (Section 3.2), enabling a deeper understanding of the different ways AI is being applied across multiple domains.
- **Block 4**: This block synthesizes the **opportunities and barriers** identified in the reviewed studies, as reported in Figure 4 (Section 3.3). These findings highlight both the potential benefits of AI applications and the barriers

This diagram provides a **step-by-step visualization** of the study’s methodological process, from bibliometric analysis to thematic categorization, comparative analysis, and the identification of emerging opportunities and challenges.

**Second Diagram (Figure 6): Connecting Discussion to Findings**

The second diagram (Figure 6) is logically connected to the first and illustrates how the study transitions from the findings of the literature review to the discussion. The sequential organization follows a structured approach:

- **Blocks 5 and 6** identify the need to bridge the gap between cutting-edge research (CER) and the current state of standardization.
- Connected to **Block 5**, two blocks highlight the contributions of CER in terms of added value and emerging challenges in Table 3 and Table 4 of Section 4.4 (**Block 7**), as well as its complementation in terms of opportunities and barriers identified in the NRR reported in Table 5 and Table 6 of Section 4.4 (**Block 8**)
- Connected to **Block 6**, **Block 9** reports in Section 4.5 the global positioning of various organizations and regulatory bodies in terms of their guidelines and recommendations.

### 4.2. Highlights

The integration of AI with interventional radiology (IR) has seen a significant increase over the last five years, largely influenced by the COVID-19 pandemic (Figure 2). This period also witnessed an expansion in review literature, which identifies established themes, highlights areas for further development, and presents emerging trends. Reviews are instrumental in providing an overview of the field, offering valuable insights into both the current state and the future potential of AI applications in IR.

Added Values from the Overview of Reviews:

*Identification of Established Areas and Themes*: Reviews have consistently highlighted AI’s growing role in improving procedural precision through integration with robotic systems for tasks like catheter manipulation, needle placement, and tumor detection. For instance, Cornelis et al. [11] emphasized that while automation and AI integration in robotic systems for image-guided interventions have advanced, no system has yet achieved full autonomy. Key metrics such as the level of autonomy in surgical robotics (LASR) and the levels of integration of advanced imaging and AI (LIAI2) scale help identify gaps and guide future developments.

Boeken et al. [12] further discussed how AI-driven robotic systems are enabling more precise and reliable procedural outcomes, especially in complex tasks like catheter manipulation and needle placement. They identified techniques such as reinforcement learning and haptic vision as vital for improving robot adaptability based on real-time feedback, advancing IR toward a model where the human role transitions to supervision rather than direct performance.

*Identification of Areas Needing Further Expansion*: While AI has made strides in several areas of IR, certain technologies, particularly those involving decision support, automation, and multimodal imaging, remain largely in the research phase. For instance, Matsui et al. [16] reviewed AI’s applications in interventional oncology, including automatic segmentation, treatment simulation, and outcome prediction. However, most AI-based methods remain experimental and are not yet fully implemented in clinical settings. Moreover, Lesaunier et al. [17] emphasized the need for further studies to enhance the reliability and clinical utility of AI, particularly in personalized treatment approaches such as individualized care planning.

A notable challenge pointed out by Zhang et al. [18] is the slow clinical adoption of AI technologies in IR, which may be attributed to regulatory barriers, ethical concerns, and the limited availability of sufficiently large and accurately labeled datasets for effective machine learning.

*Emerging Trends and Future Directions*: Reviews also highlight promising developments in AI integration, such as reinforcement learning and real-time image fusion. For example, Lee et al. [14] explored the potential of smartphones’ built-in sensors for virtual and augmented reality applications in IR, which could significantly enhance needle-based procedures like biopsy and ablation. These technologies could lead to advancements in intra-procedural guidance, enabling more precise and targeted interventions with minimal patient risk.

Additionally, the potential for AI in personalized treatment strategies is becoming increasingly apparent. As AI technologies evolve, they are expected to play a significant role in predicting treatment outcomes and optimizing care plans. Geevarghese et al. [22] highlighted AI’s ability to accelerate tumor detection and guide treatment strategies, furthering personalized medicine in IR.

*Challenges in Clinical Implementation*: Ethical considerations, regulatory barriers, and the need for reliable, clinically validated AI systems are common themes across the reviews. As noted by Warran et al. [20,21], there are concerns regarding the transparency of AI decision-making processes, and the need for appropriate validation of AI systems in clinical environments is critical. The slow adoption of AI in IR, as pointed out by Zhang et al. [18] and Mazaheri et al. [33], is another key challenge that needs to be addressed through further research, regulation, and technology deployment strategies.

*Multidisciplinary Opportunities and Future Integration*: The integration of AI, robotics, and multimodal imaging technologies holds the potential to revolutionize IR. This multidisciplinary approach will likely lead to enhanced treatment planning, real-time monitoring, and decision support systems that will complement the work of radiologists and improve patient outcomes. Posa et al. [28] emphasized the importance of engaging physicians in the development and application of AI, particularly in the field of interventional oncology, to ensure that AI technologies are effectively incorporated into clinical practice.

### 4.3. Navigating AI in Interventional Radiology: Unique Complexities and Future Directions in Comparison to Diagnostic Radiology

Radiology, including interventional radiology as a specialized branch, has seen significant advancements in its intersection with AI compared to other fields, such as digital pathology [38,39], where delays in digital standardization have also hindered AI development.

However, when we focus specifically on interventional radiology, a clear distinction emerges in how AI is being integrated compared to diagnostic radiology [40]. The role of AI differs significantly between these two branches.

In diagnostic radiology, AI primarily serves as a support tool for analyzing medical images. Advanced deep learning algorithms can detect abnormalities, such as tumors, fractures, or degenerative diseases, in images obtained from CT scans, MRI, or X-rays. This not only accelerates the diagnostic process but also reduces the risk of errors, improving both accuracy and efficiency for radiologists. Additionally, AI contributes to anatomical segmentation, image enhancement, and even disease progression prediction, further augmenting radiologists’ capabilities.

In contrast, interventional radiology presents a more complex and active role for AI. Here, AI can assist physicians during minimally invasive, image-guided procedures by improving real-time imaging, aiding in the navigation of catheters and needles, and suggesting the safest and most efficient procedural approaches. In some cases, AI even has the potential to automate certain instrument movements, reducing the margin of error and improving patient outcomes.

Furthermore, interventional radiology integrates robotics, catheterization techniques, and percutaneous dosing methods, in addition to imaging. As a result, AI in this field requires stricter safety measures, particularly due to its real-time procedural implications. These considerations extend beyond clinical safety to include cybersecurity challenges, as AI-driven interventional technologies must ensure robust protection against potential cyber threats.

All of this contributes to a delay in standardization, as the complexity of real-time AI integration in interventional radiology requires more rigorous validation, regulatory approvals, and cybersecurity measures compared to diagnostic AI applications, as highlighted in several studies analyzed [11,12,17]. Studies have pointed out that the dynamic and high-risk nature of interventional radiology (IR) procedures introduces additional challenges in ensuring the reliability and safety of AI systems during live interventions [13,19]. The need for thorough validation in clinical environments, where AI-guided systems interact with multiple variables such as patient anatomy and real-time imaging, makes the integration process more complex than in diagnostic radiology, where AI typically works with static images and less variable conditions [17,25]. Moreover, ensuring that AI systems meet stringent regulatory and cybersecurity standards is essential to protect patient safety and prevent potential breaches in sensitive healthcare data, a concern raised in multiple studies [27,31].

On the comparative side of scientific production, a search on PubMed using the search key 3 in box 1 [41] reveals a substantial surge in interest in AI in diagnostic radiology starting in 2018 (with the exception of a few sporadic initiatives). A total of 126 studies were identified, 111 of which were published in the past five years, accounting for 88.1% of the total, with 34 of these being review articles. This dissemination activity is comparable to that in interventional radiology, which saw 114 studies, 109 of which were published in the last five years. Therefore, research trends show a significant rise in AI publications in both diagnostic and interventional radiology, especially in the past five years and during the pandemic. The high number of review articles reflects growing interest in AI applications, with both fields demonstrating similar dissemination patterns.

What sets them apart is the differing vision on the future development of interventional radiology [42]. On one hand, there is a strong belief within the scientific community that the future of interventional radiology should remain closely aligned with diagnostic radiology, fostering collaboration and integration [43]. On the other hand, some experts argue for a more independent trajectory for interventional radiology, emphasizing its distinct role and specialization [44]. This divide reflects ongoing debates on how best to position the field in relation to other radiological practices.

### 4.4. AI in Interventional Radiology: Pioneering Studies Tackling Challenges, Exploring Opportunities, and Driving Key Innovations

Recent studies [45,46,47,48,49,50,51,52,53,54,55,56,57,58,59,60,61,62] have also been considered (as well as valuable editorial mapping of the route) to explore how they align with the challenges identified in the overview of reviews. The integration of AI into IR offers promising advancements, but it also brings a set of significant challenges that need to be addressed thoughtfully. The potential for AI to enhance precision, optimize patient outcomes, and revolutionize treatment planning is undeniable. However, this integration also requires careful consideration of multiple factors to ensure its success and avoid unintended consequences.

One of the first challenges lies in data availability, particularly when it comes to deep learning (DL) models in fluoroscopy-guided interventions (FGIs). Obtaining large datasets of labeled clinical images for training AI models has traditionally been difficult. To address this, the use of synthetic data has been proposed, generated from advanced imaging techniques like CT angiography. While this approach promises to alleviate the data scarcity issue, it raises questions about the accuracy and reliability of synthetic images. AI models trained on synthetic data must be rigorously validated to ensure they can effectively support real-world clinical decision-making [46].

Another important challenge is the acceptance of AI in clinical practice, particularly in areas where it directly impacts patient communication and informed consent. Studies have shown that while AI tools, such as ChatGPT, can provide accurate and understandable information, they often fall short in offering comprehensive responses. This raises concerns among clinicians about relying on AI-generated outputs for critical tasks such as discussing risks and benefits with patients. Ensuring AI systems can deliver more than just surface-level answers and provide in-depth, context-sensitive information is crucial before they can be fully embraced in clinical settings [47].

Moreover, the integration of AI in IR demands a focus on continuous education and training. As AI technologies evolve rapidly, IR professionals must keep pace with these advancements, equipping themselves with the skills necessary to harness the full potential of these innovations. The challenge, however, is not just about acquiring technical knowledge but also about maintaining an ergonomic approach to minimize the risk of fatigue or strain from using new AI tools. Furthermore, there is a need to consider the economic implications of adopting AI. Innovations must not only improve patient care but also be economically viable to ensure widespread adoption and sustainability within healthcare systems [48]. AI’s potential to disrupt the field of IR brings both opportunities and risks. While these innovations can greatly improve efficiency and patient outcomes, they also present a challenge in adapting to and managing technological disruptions. Interventional radiologists must not only accept these changes but actively engage in shaping how AI is developed, trained, and implemented. This requires leadership, collaboration, and an ongoing commitment to understanding the broader implications of AI on clinical practice and patient care [49]. The integration of AI into IR, while transformative, requires careful navigation to address challenges related to data, clinical practice, education, and economic feasibility. As AI continues to evolve, interventional radiologists must lead this change, balancing technological innovation with a focus on improved patient care and operational sustainability [50]. While technologies such as large language models (LLMs) and robotic systems show promise in enhancing procedural precision and patient understanding, there are still obstacles that need to be addressed for optimal clinical implementation.

One of the main challenges is the dependency on linguistic context, as demonstrated in a study comparing ERNIE Bot and ChatGPT in answering questions about liver cancer interventional radiology. Both technologies showed potential, but their performance varied significantly depending on the language. ERNIE Bot outperformed ChatGPT in the Chinese context, while ChatGPT performed better in English [51]. This discrepancy suggests that LLMs must be adapted not only to the language but also to the specific cultural and healthcare contexts to be truly effective. Improving the ability of AI models to understand and respond accurately to medical questions in different languages and contexts remains a key challenge.

Furthermore, while robotic systems have demonstrated improvements in precision and efficiency during CT-guided procedures, there are still substantial hurdles to overcome. A robotic system designed for lesion detection and path planning showed reduced insertion time and improved accuracy compared to traditional freehand techniques [52]. However, integrating advanced robotic systems into clinical procedures requires overcoming technological challenges related to calibration, safety, and reliability of the devices. Additionally, the complexity of adapting robots to various patient types and clinical situations presents an additional obstacle to their widespread adoption.

Another challenge concerns the use of AI to simplify radiology reports and make information more accessible to patients. Although radiologists recognize the potential of AI in report simplification, most of them indicated that human oversight is necessary. In one study, the majority of radiologists expressed a preference for AI-generated simplifications to be reviewed by professionals [54]. This highlights a reluctance to fully rely on AI without adequate supervision, especially in a field where precision is crucial. The challenge is to find a balance between automation and human control that ensures optimal quality and safety.

Additionally, while deep learning models have shown high accuracy in identifying anatomical locations in digital subtraction angiography (DSA), further refinement is needed. Despite improving location classification in angiographic sequences, challenges remain related to reliability and the ability to handle complex or atypical cases [53]. The difficulty in managing non-standard or uniquely complex cases is one of the main barriers to the widespread adoption of AI in interventional radiology.

AI has also shown potential in automating collimation in interventional radiology, an area that is often underutilized due to its complexity. Collimation is essential for improving image quality and minimizing radiation exposure, but it requires manual adjustments that can be time-consuming and vary widely across procedures and operators. A tunable algorithm for automatic collimation, as demonstrated in a study, showed promising results in reducing radiation exposure while meeting the varying preferences of interventionalists [55]. Despite its potential, the challenge lies in ensuring that AI-driven solutions can be seamlessly integrated into fast-paced, high-stakes environments without disrupting the workflow or procedure quality.

AI’s role in advancing diagnostic and therapeutic techniques, such as in the analysis of parapharyngeal space (PPS) lesions, has also shown promise. Advanced imaging, supported by AI algorithms, allows for precise lesion diagnosis and planning of interventions, such as CT-guided biopsies [56]. However, AI’s ability to accurately differentiate between pathologies still requires further validation, especially in real-world clinical settings. While AI has contributed significantly to tumor segmentation, lymph node metastasis detection, and pathological classification, challenges remain in its application across all head and neck pathologies, particularly in rare or complex cases. In the specific field of Interventional Oncology (IO), there have been numerous advancements in recent years, showing immense potential for transforming cancer treatment and management. However, IO faces unique challenges as it bridges the gap between diagnostic radiology and clinical oncology. While promising, these advancements must overcome difficulties in integration with clinical workflows, as well as address the accuracy of AI and robotic systems in highly specialized cancer treatments [45].

The use of LLMs like GPT-4 and Claude-3-Opus to simplify IR reports has shown promising results in terms of readability and clinical relevance. However, errors that could undermine patient trust in the information provided have been observed [57]. Trust-breaking errors, such as those that may lead to confusion or misunderstanding, are a major concern when using AI in healthcare. To be adopted in real-world clinical settings, these technologies must be refined to minimize errors and ensure clear, accurate communication. Further recent studies highlight progress in areas such as immunotherapy, patient education, safety data analysis, exam preparation, and advanced imaging techniques. In cancer treatment, immunotherapy, particularly intratumoral approaches, represents a paradigm shift. By integrating machine learning algorithms and radiomics, AI allows for the analysis of complex imaging data to refine diagnoses, predict treatment responses, and tailor therapeutic strategies. These advancements have the potential to improve patient survival and quality of life while minimizing side effects, potentially reshaping clinical guidelines. However, further clinical studies are needed to confirm the efficacy of these promising approaches [58]. Patient education is another area where AI shows great promise. The use of GPT-4 to generate easily understandable educational material for interventional radiology procedures has yielded positive results. The AI-generated instructions were found to be more readable and accessible compared to traditional sources, improving patient comprehension and satisfaction. This approach can significantly enhance health literacy and patient-centered care, making it easier for patients to understand their treatment options and procedures. However, further work is needed to refine these materials for more complex procedures and to ensure consistency and accuracy across different types of interventional radiology treatments [59].

AI is also making strides in safety data analysis in IR. GPT-4 has been used to classify and summarize microwave ablation device safety event data accurately. The AI model demonstrated high accuracy in categorizing failures and generating meaningful summaries, helping clinicians interpret large volumes of safety data more effectively. This ability to streamline data processing could improve the efficiency and safety of IR procedures. Nevertheless, additional validation across a wider variety of devices and event types is necessary to improve the generalizability of these findings and address the limitations of the model when dealing with more diverse datasets [60].

Moreover, GPT-4 has been tested in medical exam preparation, particularly for the European Board of Interventional Radiology (EBIR) exam. The AI was able to generate exam questions at various difficulty levels, assisting medical students and professionals at different stages of their careers. The results showed that GPT-4 could help train medical students and interventional radiologists by generating relevant exam items, thereby aiding in exam preparation and professional development. One area of improvement, however, lies in refining the AI’s ability to generate items with varying degrees of difficulty to better match the knowledge and experience levels of a diverse audience, ensuring that it meets the educational needs of both beginners and experts [61].

In advanced imaging, AI-enhanced techniques have been developed to generate synthetic contrast-enhanced images from non-contrast CT scans, particularly in the context of renal cancer treatments. This approach reduces the reliance on iodine-based contrast agents, which can be associated with adverse reactions and higher radiation doses. The AI models demonstrated competitive image quality and improved performance in downstream tasks, such as image segmentation, offering significant benefits for interventional procedures. While promising, these techniques would benefit from further refinement in handling various anatomical structures and improving the accuracy of image enhancement across diverse patient populations [62].

Table 3 highlights key advancements in AI technologies in interventional radiology. These innovations are improving precision, simplifying processes, and advancing diagnostic and therapeutic practices. However, challenges still remain for full clinical implementation.

**Table 3:** Key advancements in AI applications in interventional radiology.

Advancements	Description	Related Studies
AI Integration for Improved Precision	AI has demonstrated significant potential in enhancing precision, optimizing patient outcomes, and revolutionizing treatment planning in IR.	[46,47,48,49,50]
AI in Robotic Systems	Robotic systems have shown improvements in precision and efficiency, particularly in lesion detection and path planning during CT-guided procedures.	[52]
AI Simplification of Radiology Reports	AI simplifies complex radiology reports, improving accessibility and readability for both clinicians and patients, but requires oversight to ensure quality.	[54]
AI in Parapharyngeal Space (PPS) Lesion Diagnosis	AI-assisted imaging has shown potential for precise diagnosis of PPS lesions and improved intervention planning, such as for CT-guided biopsies.	[56]
Advancements in Interventional Oncology (IO)	IO innovations are transforming cancer treatment, bridging diagnostic radiology and oncology, though challenges remain in integration and accuracy.	[45]
AI in IR Report Simplification and Accuracy	Large language models like GPT-4 and Claude-3-Opus simplify IR reports for better readability, though errors need to be minimized to ensure trust and clarity.	[57]
Linguistic Context Dependency	AI’s performance varies based on language and cultural context, impacting its application in liver cancer IR.	[51]
Deep Learning Model Refinement	While deep learning models aid anatomical identification, they face challenges in handling complex or atypical cases.	[53]
Automating Collimation	AI-driven collimation algorithms reduce radiation exposure and enhance image quality, but integrating them into fast-paced clinical workflows remains difficult.	[55]
Immunotherapy and Intratumoral Approaches	AI, through machine learning and radiomics, refines diagnosis and predicts treatment responses, optimizing personalized care in oncology.	[58]
Patient Education and Communication	GPT-4 aids in simplifying explanations of IR procedures, improving patient understanding and health literacy.	[59]
AI in Safety Data Analysis	GPT-4 categorizes and summarizes safety event data from IR devices, streamlining data analysis for improved patient safety.	[60]
Training and Exam Preparation	GPT-4 generates exam items for medical professionals, supporting EBIR exam preparation and training for various expertise levels.	[61]
Improved Imaging Techniques	AI-enhanced imaging generates synthetic contrast-enhanced images from non-contrast CT scans, improving image quality and reducing contrast agent use.	[62]

Table 4 outlines the main challenges in integrating AI into interventional radiology. These challenges range from data availability to the need for continuous education and training for professionals. Addressing these issues is crucial for the successful application of AI in clinical settings.

**Table 4:** Challenges in the integration of AI in interventional radiology.

Challenges	Description	Related Studies
Data Availability	Difficulty in obtaining large datasets of labeled clinical images for AI model training, especially for fluoroscopy-guided interventions (FGIs). Synthetic data could help, but it needs rigorous validation.	[46]
Acceptance in Clinical Practice	AI’s ability to provide comprehensive, context-sensitive information for patient communication and informed consent remains a challenge. AI-generated outputs need more than surface-level responses.	[47]
Continuous Education and Training	IR professionals must keep up with rapidly evolving AI technologies, ensuring they can use these innovations effectively and ergonomically. Economic feasibility of AI adoption is also a key concern.	[48]
Linguistic Context Dependency	AI’s performance varies based on language and cultural context. For instance, ERNIE Bot and ChatGPT had differing performances in liver cancer IR depending on the language (Chinese vs. English).	[51]
Robotic Systems Integration	While robotic systems enhance precision in CT-guided procedures, challenges related to calibration, safety, and adaptability to different patients remain.	[52]
Radiology Report Simplification	AI can simplify radiology reports, but human oversight is often preferred by radiologists to ensure accuracy and quality, reflecting hesitance to fully trust AI.	[54]
Deep Learning Model Refinement	While deep learning models are useful for anatomical identification, issues persist with handling complex or atypical cases, requiring further refinement.	[53]
Automating Collimation	AI-driven collimation algorithms can reduce radiation exposure and improve image quality, but their integration into fast-paced clinical workflows remains challenging.	[55]
AI in Parapharyngeal Space (PPS) Lesions	AI shows potential in lesion diagnosis and intervention planning but needs further validation to ensure accuracy, especially in complex or rare cases.	[56]
Trust and Accuracy of AI Systems	While LLMs like GPT-4 and Claude-3-Opus show promise in simplifying IR reports, trust-breaking errors need to be addressed for clinical implementation.	[57]
Advancements in Interventional Oncology (IO)	IO innovations are transforming cancer treatment, bridging diagnostic radiology and oncology, though challenges remain in integration and accuracy.	[45]
Navigating Technological Change in IR	The integration of AI in IR requires leadership, collaboration, and an ongoing commitment to understanding its broader implications for patient care and clinical practice.	[50]
Data Quality and Consistency in Patient Education	Despite positive results in using AI for patient education, challenges remain in ensuring that AI-generated content is accurate, consistent, and suitable for all patients.	[58]
AI Reliability in Data Classification	AI models like GPT-4 for safety event data classification are effective, but there are concerns about the model’s reliability when handling complex or rare data.	[59]
AI Integration into Medical Education	AI’s role in medical exam preparation is promising, yet further validation is required to ensure that AI-generated questions are clinically relevant and effective for training purposes.	[60]
Image Quality and Safety in Advanced Imaging	While AI can generate synthetic contrast-enhanced images, ensuring their quality and clinical validity without causing patient harm remains a significant challenge.	[61]
Safety and Efficacy of AI in High-Stakes Procedures	AI models for synthetic contrast imaging are beneficial, but there is a need to address concerns about safety, accuracy, and clinical efficacy in high-stakes procedures like renal cancer treatments.	[62]

It is particularly interesting to observe how the contributions of these cutting-edge research studies align, in some cases, directly with the opportunities and barriers outlined in Figure 4. This convergence highlights the relevance of recent advancements in artificial intelligence (AI) to the broader discussion on its integration into interventional radiology.

Table 5 and Table 6 provide a structured comparison between the cutting-edge research (CER) and the narrative review of reviews (NRR), mapping their respective contributions to the identification of key opportunities and challenges. By analyzing these contributions side by side, it becomes possible to assess how different methodological approaches—one focused on direct evidence synthesis and the other on a broader synthesis of existing reviews—complement each other in providing a comprehensive perspective on AI implementation in interventional radiology.

This comparison underscores the need for a balanced approach that not only identifies the technical and clinical advantages of AI integration but also acknowledges the potential barriers related to regulatory frameworks, clinical acceptance, workflow adaptation, and long-term sustainability. By leveraging insights from both CER and NRR methodologies, we can obtain a more nuanced understanding of how AI can be effectively deployed while addressing the critical challenges associated with its adoption.

**Table 5:** Opportunities from NRR and contributions from CER.

Opportunities form NRR	Description of Contribution from CER	References
1. Improving Accuracy and Efficiency	AI plays a key role in enhancing diagnostic accuracy and operational efficiency within interventional radiology (IR). Deep learning models, for example, have been shown to improve the accuracy of anatomical location classification in imaging techniques like digital subtraction angiography (DSA). Additionally, the integration of AI into robotic systems for lesion detection and path planning helps ensure more precise targeting, reducing errors, and increasing procedural success rates. These advancements collectively improve patient outcomes and workflow efficiency.	[52,53]
2. Personalization of Treatment	AI is significantly advancing personalized treatment planning, particularly in interventional oncology (IO). AI’s ability to improve tumor segmentation and detect lymph node metastasis allows for more tailored and accurate treatment strategies that are customized to individual patient profiles. For instance, AI is used to refine treatment planning for procedures like CT-guided biopsies, ensuring better-targeted interventions that align with the patient’s specific condition. This approach is vital for enhancing patient care and improving outcomes, especially in complex or rare cases.	[56]
3. Enhanced Intra-Procedural Guidance	AI is crucial for improving intra-procedural guidance in IR. For example, robotic systems used in procedures like CT-guided interventions have shown improvements in precision and accuracy, enabling real-time decision-making and guidance. AI algorithms that automatically control collimation during procedures also contribute by reducing radiation exposure while maintaining high image quality. This capability is crucial for ensuring both patient safety and procedural success, making AI an essential tool in modern IR workflows.	[52,55]
4. Automation and Robotics	The integration of AI-driven automation and robotic systems significantly enhances the accuracy, efficiency, and precision of interventional procedures. Robotic assistance not only improves procedural accuracy but also reduces clinician fatigue, particularly when performing repetitive or complex tasks. Moreover, AI’s role in automating tasks like report generation or collimation streamlines workflows, allowing healthcare professionals to allocate more time for critical decision-making and patient care. These advancements support the optimization of clinical outcomes in IR.	[52]
5. Multimodal Integration	AI’s capacity to integrate different modalities, such as imaging, robotics, and real-time data analysis, is revolutionizing IR. Recent innovations, such as generating synthetic contrast-enhanced images from non-contrast CT scans, reduce reliance on contrast agents while maintaining high diagnostic quality. By improving imaging accuracy and optimizing procedural planning, AI-driven multimodal integration enhances treatment strategies and reduces patient exposure to harmful substances, making it a game-changer in the field.	[62]

**Table 6:** Barriers from NRR and contributions from CER.

Barriers from NRR	Description of Contribution of CER	References
1. Ethical Considerations	Ethical challenges are a major barrier to AI adoption in IR. Concerns around data privacy, patient consent, and potential biases in AI models must be addressed to ensure that AI systems are ethically deployed. Additionally, the use of AI tools for tasks such as explaining procedures or risks to patients raises questions about whether these systems can provide contextually accurate and sensitive information. Clinicians may hesitate to fully rely on AI, especially when it comes to explaining complex medical situations to patients.	[47]
2. Regulatory Barriers and Intellectual Property	Regulatory hurdles pose significant challenges to the integration of AI in clinical practice. AI technologies require rigorous validation and approval processes to ensure their safety and effectiveness. However, the rapid pace of AI development often outstrips existing regulatory frameworks, causing delays in clinical adoption. Furthermore, intellectual property issues related to AI models and the sharing of new technologies complicate collaboration and innovation in healthcare. These obstacles must be addressed to facilitate the broader use of AI in interventional radiology.	[46]
3. Reliability and Clinical Utility	The reliability and clinical utility of AI systems remain significant barriers to their widespread adoption. While AI models have demonstrated promising results in controlled environments, their real-world performance may be inconsistent due to variations in data quality, model training, and the complexity of clinical cases. For example, AI models trained on synthetic data may not perform as effectively in real-world clinical scenarios, particularly in atypical or complex cases. This raises concerns among clinicians, who may be reluctant to trust AI outputs without human oversight, especially in critical applications like interventional procedures.	[46,54]
4. Slow Adoption	Slow adoption of AI technologies remains a key barrier in interventional radiology. While there is significant interest in AI’s potential, many professionals are hesitant to integrate it into their practices. Concerns about the clinical utility of AI, the need for continuous training, and the disruption of established workflows all contribute to this resistance. Additionally, the financial and logistical challenges involved in adopting AI technologies, such as the cost of new equipment and system integration, hinder the widespread implementation of AI in clinical settings.	[48]

### 4.5. Emerging Directions in Interventional Radiology: AI Integration and the Path to Global Harmonization

Interventional radiology devices, from a medical device perspective, are complex systems with a high degree of interoperability. These devices can include, in addition to radiative imaging systems, components of robotics, catheterization, and others. Even without AI, they already have a highly intricate certification and integration process within the healthcare domain. A key example is cybersecurity, which has significant implications for patient safety, much more so than database management. In interventional radiology (IR), a cyberattack—whether altering the dose, compromising robotics, or other aspects—directly impacts patient safety and can cause harm.

The addition of AI to IR, which already brings its own challenges in integration, and even more so in the healthcare domain, introduces further questions and problems, on top of the well-known ones. Therefore, the role of national and international bodies, alongside national and international scientific societies, is crucial. National bodies must aim to establish as uniform and comprehensive an approach as possible, ideally unified, rather than dissimilar, as is currently the case for medical devices in general. Scientific societies must take concerted, impactful actions to develop broad and inclusive guidelines in this area. The works by Warren et al. [20,21] are crucial in guiding the integration of AI into IR by addressing key challenges and proposing structured solutions. They focus on the importance of clinician education in AI, the classification of AI models based on complexity, and the assessment of AI applications across different procedural stages (pre-, intra-, and post-procedural).

Additionally, these studies emphasize the need for rigorous risk evaluation, outlining a hierarchy of potential harms and providing a checklist for AI deployment in clinical IR environments. Their direction aligns with the broader goal of establishing clear regulatory frameworks and safety guidelines to ensure the responsible and effective implementation of AI in IR.

An important future direction is to focus on the two strategic points: medical devices (MD) and guidelines, which will involve scientific societies, certifying bodies, and national or supranational legislative entities. This is also a strongly suggested future research direction, to observe what is happening internally. Achieving uniformity at the international level will be the next step. In fact, the integration of artificial intelligence (AI) into interventional radiology is gaining increasing attention, partly due to the growing use of advanced tools that combine AI with medical devices to support professionals during image-guided procedures. While much of the international guidelines focus primarily on diagnostic radiology, many of the principles developed by professional bodies and regulatory agencies can also be directly applied to interventional radiology, which, as a part of radiology, shares many challenges and opportunities in adopting AI technologies.

For instance, the Radiological Society of North America (RSNA) has developed a comprehensive strategy for AI use in radiology, which, although primarily focused on diagnostics, offers valuable insights for interventional radiology [63]. The RSNA emphasizes the importance of continuous clinical validation, balanced AI integration into workflows, and the monitoring of AI tool performance. One of the main recommendations is to use AI as support, not as a substitute for healthcare professionals—an essential principle for interventional radiology, where AI can optimize procedural precision but should always be used under expert supervision.

The American College of Radiology (ACR), in collaboration with the RSNA, has produced guidelines for implementing AI tools in radiology [64]. While the focus is on diagnostic applications, these guidelines offer relevant points for interventional radiology, particularly regarding the need for proper professional training to safely use AI in clinical procedures. The ACR stresses that AI adoption should be accompanied by continuous education for radiologists, ensuring they are prepared to adapt to technological changes while also managing risks, such as potential loss of practical skills in the face of automation.

The Society of Radiographers (SoR) [65] has also developed guidelines for AI use in diagnostics, which, although centered around clinical imaging, provide valuable suggestions on how AI should be integrated while maintaining human control. In interventional radiology, this principle of human oversight is crucial, as AI tools used for image guidance during procedures must always be monitored by qualified professionals to prevent potential errors during delicate interventions.

The Royal College of Radiologists (RCR) [66], with its recommendations for AI integration into radiological workflows, emphasizes the need for a gradual introduction of AI to avoid disruptions to existing workflows and ensure patient safety. This approach is particularly relevant in interventional radiology, where AI integration could influence the efficiency of real-time procedures, but it must be done in a way that does not compromise the quality of clinical decision-making.

Regulatory bodies like the U.S. Food and Drug Administration (FDA) have also played a critical role in shaping the landscape of AI in medical devices, including in interventional radiology [67,68,69,70]. The FDA has approved several AI-based software systems aimed at assisting interventional radiologists during image-guided procedures. These approvals underscore the need for regulatory oversight in AI tool deployment, ensuring that they meet high standards for safety and effectiveness before being used in clinical practice. The FDA’s focus on Software as a Medical Device (SaMD) provides a clear regulatory pathway for AI tools used in IR, ensuring that these tools are developed with the same scrutiny as any other medical device.

The regulatory guidance from the FDA is complemented by ongoing developments in the European Union, where lawmakers are refining policies under the EU AI Act [71]. This legislation aims to regulate the development and use of AI across sectors, including healthcare. By adhering to these international guidelines, both diagnostic and interventional radiology practices can ensure that AI is used in a safe, effective, and ethically responsible manner. An example of movement is the ESR initiative [72] producing a statement on how the EU AI Act applies to radiology and therefore also to IR. It outlines key policies and offers recommendations to policymakers and professionals for effectively implementing the legislation, addressing gaps and uncertainties. It covers areas such as AI literacy in radiology, classification of high-risk AI systems, data governance, transparency, human oversight, quality management, obligations of AI deployers, regulatory sandboxes, post-market monitoring, information sharing, and market surveillance. The statement highlights ESR’s commitment to supporting the safe and effective integration of AI in radiology to benefit patients across Europe.

### 4.6. Limitations

This narrative review follows a structured methodology with well-defined inclusion and exclusion criteria, ensuring a focused and high-quality synthesis of available literature. While the exclusion of conference proceedings means that some emerging research or preliminary developments may not be captured, this approach prioritizes studies that have undergone rigorous peer review, enhancing the reliability of the findings. Additionally, by focusing on internationally published literature in English, the review ensures broad applicability and comparability across different healthcare settings. However, this may result in the omission of region-specific insights or localized best practices, highlighting an opportunity for future research to explore diverse clinical approaches and treatment protocols in various cultural and healthcare contexts.

## 5. Conclusions

The integration of artificial intelligence (AI) into interventional radiology (IR) presents both significant opportunities and challenges that will undoubtedly shape the future of this field. This technology has the potential to greatly enhance the precision, efficiency, and personalization of IR procedures. By automating complex tasks, such as catheter manipulation and needle placement, it improves procedural accuracy and reduces variability. Furthermore, its ability to integrate multiple imaging modalities supports personalized patient management, optimizing treatment plans and outcomes. Intra-procedural guidance also benefits from these advancements, enhancing precision through technologies like needle tracking and real-time image fusion.

As AI continues to evolve, so too does the potential for robotics and automation in IR. AI-guided robotic systems are set to play an increasingly autonomous role, transforming the function of interventional radiologists from active procedure performers to supervisors of intelligent systems. However, full autonomy in these systems has not yet been achieved, and there remain significant areas for further development and integration. Despite these promising advancements, challenges remain.

IR devices represent a highly complex and integrated system within healthcare. These devices, which include radiative imaging systems, robotics, catheterization, and other advanced technologies, require a detailed certification and integration process. This complexity is further amplified by the inclusion of AI, which introduces new challenges and considerations. As this technology becomes more deeply integrated into IR, it raises numerous questions that extend beyond traditional device integration concerns. The role of national and international bodies, along with scientific societies, is crucial in navigating this evolving landscape. These organizations must work together to establish uniform and comprehensive regulatory frameworks that ensure safety and efficacy across different healthcare systems.

Moving forward, a key direction for IR will focus on developing clear, standardized guidelines for both medical devices and AI applications. Collaboration between certifying bodies, scientific societies, and legislative entities will be essential in ensuring global uniformity. Ongoing efforts, such as the EU AI Act, highlight the importance of addressing AI-specific challenges. In the context of radiology, initiatives like those from the European Society of Radiology (ESR) that align with the EU AI Act—focusing on transparency, data governance, human oversight, and post-market monitoring—serve as exemplary steps in the right direction.

**The integration of these technologies makes interventional radiology more personalized**, **patient-centered**, **and safe**. With concerted efforts, the integration of advanced technologies in IR can proceed with the necessary safeguards, ultimately benefiting patient outcomes and advancing the field responsibly.

## Data Availability

Not applicable.
